# HIF-1-induced mitochondrial ribosome protein L52: a mechanism for breast cancer cellular adaptation and metastatic initiation in response to hypoxia

**DOI:** 10.7150/thno.57804

**Published:** 2021-05-25

**Authors:** Xinyan Li, Mengshen Wang, Su Li, Yuqiong Chen, Mozhi Wang, Zhonghua Wu, Xiangyu Sun, Litong Yao, Haoran Dong, Yongxi Song, Yingying Xu

**Affiliations:** 1 Department of Breast Surgery, The First Affiliated Hospital of China Medical University, Shenyang, Liaoning, China.; 2 Department of Cardiology, Shanghai Institute of Cardiovascular Diseases, Zhongshan Hospital, Fudan University, Shanghai, China.; 3 Department of Cardiology, The First Affiliated Hospital of China Medical University, Shenyang, Liaoning, China.; 4 Department of Surgical Oncology and General Surgery, Key Laboratory of Precision Diagnosis and Treatment of Gastrointestinal Tumors, Ministry of Education, The First Affiliated Hospital of China Medical University, Shenyang, Liaoning, China.

**Keywords:** Breast cancer, Hypoxia, Mitochondrial ribosome, Mitophagy, Metastasis

## Abstract

**Background:** Hypoxia is a hallmark of the physical microenvironment of solid tumors. As a key factor that regulates tumor development and progression, hypoxia can reprogram the expression of multiple genes, whose biological function and molecular mechanism in cancer remain largely unclear. The mitochondrial ribosome protein family consists of nuclear-encoded mitochondrial proteins that are responsible for protein synthesis in the mitochondria.

**Methods:** A high-throughput RNA sequencing assay was carried out to identify differentially expressed mRNAs between breast cancer tissues and adjacent normal tissues as well as breast tumors with metastasis and those without metastasis. Our clinical samples and TCGA database were analyzed to observe the clinical value of mitochondrial ribosome protein L52 (MRPL52) in human breast cancer. Potent hypoxia response elements in the promoter region of MRPL52 were identified and validated by chromatin immunoprecipitation and luciferase reporter assays. Functional experiments were performed using breast cancer cell lines with MRPL52 ectopic expression and knockdown cultured in a 20% or 1% O_2_ environment.

**Results:** MRPL52 expression was upregulated in human breast cancer and was significantly associated with aggressive clinicopathological characteristics and a higher metastatic risk of breast cancer patients. We found that the overexpression of MRPL52 in breast cancer is induced by hypoxia-inducible factor-1 in response to hypoxic exposure. The role of MRPL52 in suppressing apoptosis and promoting migration and invasion of hypoxic breast cancer cells was demonstrated by our experimental evidence. Mechanistically, MRPL52 promoted PTEN-induced putative kinase 1 /Parkin-dependent mitophagy to remove oxidatively damaged mitochondria and prevent uncontrolled reactive oxygen species (ROS) generation, thus repressing activation of the mitochondrial apoptotic cascade. Additionally, MRPL52 augmented epithelial-mesenchymal transition, migration and invasion of hypoxic breast cancer cells by activating the ROS-Notch1-Snail signaling pathway. Benefited from this bidirectional regulatory mechanism, MRPL52 is responsible for maintaining ROS levels in a window that can induce tumorigenic signal transduction without causing cytotoxicity in hypoxic breast cancer cells.

**Conclusions:** This work elucidates the molecular mechanism by which MRPL52 mediates hypoxia-induced apoptotic resistance and metastatic initiation of breast cancer, and provides new insights into the interplay between cancer and the tumor microenvironment.

## Introduction

Breast cancer (BC) has always been a major issue threatening public health globally. Recent data shows that BC has replaced lung cancer as the most commonly diagnosed cancer worldwide (IARC, 2020). A major cause of mortality in BC is metastasis, which accounts for more than 90% of BC-related deaths 1, 2. The high heterogeneity of the tumor microenvironment may alter the cellular composition and metabolic characteristics of cancer cells, the mechanism of which still requires further exploration 3, 4. Hypoxia, a condition involving deprivation of oxygen (< 2% O_2_) for organisms, tissues or cells, is a fundamental and life-threatening biological phenomenon. Occurring in 90% of solid tumors, hypoxia plays a key role in inducing malignant tumor phenotypes and elevating chemotherapeutic resistance, which has been linked to an increased metastatic potential 5-8. Hypoxia inducible factors (HIFs) are master hypoxic responsive factors that function as transcription factors to bind and activate the hypoxia response elements (HREs) of various target genes, which participate in epithelial-mesenchymal transition (EMT), angiogenesis, autophagy and metabolic alterations 9-13. Indeed, overexpression of HIF-1α has been detected to be significantly associated with dismal prognosis in BC patients 14-16. Therefore, further exploration of the molecular mechanisms coupling hypoxia to metastasis in a HIFs-dependent manner will deepen our understanding of microenvironment-tumor interactions and open new opportunities for BC management and treatment. Since mitochondria is the main organelle that affected by altering microenvironmental oxygen and have been linked to tumor development by increasing numbers of studies 17-22, there may be potential mediators of hypoxia-induced BC progression and metastasis in mitochondria-related genes.

Approximately 1/4 of nuclear-encoded mitochondrial proteins contribute to regulating mitochondrial gene (mtDNA) expression in mammals 23. The mitochondrial ribosome protein (MRP) family contains 30 small mitoribosomal proteins (MRPSs) and 52 large mitoribosomal proteins (MRPLs) which are encoded by nuclear genes 24, 25. Human mitoribosomes are responsible for the synthesis of 13 mtDNA-encoded proteins that constitute mitochondrial electron transport chain (ETC) complex Ⅰ and Ⅲ-Ⅴ 26, 27. Therefore, MRP family proteins are possible regulators of mtDNA translation and function during cell development. In recent years, dysregulation of MRP family genes has been found in various cancers. The upregulation of nearly 40 MRPs has been found in human BC cells, but not in adjacent stromal cells 28. Microenvironmental lactate could downregulate the expression of MRPL13, leading to impaired mitochondrial oxidative phosphorylation (OXPHOS) and increased invasiveness of hepatoma via the reactive oxygen species (ROS)-Claudin-1 pathway 29. MRPL33 and its splicing regulator promote growth and suppress apoptosis of colorectal cancer cells 30. MRPS23 acts as a key regulator of tumor proliferation in hepatocellular carcinoma and BC 31-33, and participates in metastatic phenotypes of cervical cancer 34. MRPL35 is highly expressed in colorectal cancer and its knockdown leads to ROS accumulation, loss of cell proliferation, G2/M arrest, apoptosis and cell death 35. These works indicated that MRP proteins are important regulators of nearly every aspect of cancer cells. Tumor cells initiate alterations in the expression of various mitochondria-related genes in response to hypoxia, which mediate metabolic reprogramming, adaptation and progression of cancer 36-40. Nevertheless, the expression pattern and functional mechanism of MRP family genes in BC under hypoxic conditions have not yet been investigated.

ROS are a main byproduct of the OXPHOS reaction that occurs in the mitochondria of mammalian cells. At normal cellular levels, ROS regulate a wide range of cellular responses, including growth, differentiation, ageing, transcription factor activity, and inflammatory reactions 41-47. Nevertheless, abnormal generation of ROS has been discovered to be involved in various diseases, such as cancer, cardiovascular disease, diabetes and skin disorders 45, 48-52. Deficiency of O_2_ in the tumor microenvironment is one of the principal causes of ROS overproduction in cancer cells. Since O_2_ is the electron recipient of the ETC, hypoxia can lead to an imbalance in electron transfer; thus, more electrons leak into intermembrane spaces and finally trigger increased production of superoxide anion (O_2_^-^) 49, 53, 54. Most O_2_^-^ is dismutated to hydrogen peroxide (H_2_O_2_) by superoxide dismutases in the mitochondrial matrix or intermembrane space. H_2_O_2_ is highly diffusible and enters the cytoplasm as a second messenger. Moderately increased ROS in hypoxic cancer cells could stabilize HIF-1α and mediate various tumorigenesis-associated signaling pathways, including AKT, NF-κB, AMPK and Notch 55-60. Nevertheless, a vicious cycle between mitochondria damage and ROS generation could threaten the survival of hypoxic cancer cells. ROS produced in the mitochondrial matrix lead to damage to the mitochondrial membrane and DNA, and the damaged mitochondria in turn drive sustained production of ROS to a toxic level 61. When oxidatively damaged mitochondria over-accumulate, they may ultimately swell, rupture, lose membrane potential (Δψ_m_) and release Cytochrome c (Cyt c) to trigger programmed cell death 62. Thus, removing dysfunctional or damaged mitochondria is essential for maintaining redox balance and survival in cancer cells exposed to hypoxia. Autophagy is a main mechanism by which cells remove damaged cellular components. Mitophagy is a specific type of autophagy that occurs in mitochondria and has been shown to control ROS production and support cancer cell survival by removing damaged mitochondria 11, 63, 64.

Here, we first identified MRPL52 as a hypoxia-regulated protein in a HIF-1-dependent manner; its upregulation was significantly correlated with higher metastatic risk and poorer clinicopathological characteristics in a set of human BC specimens. Importantly, our work describes a bidirectional regulatory effect of MRPL52 on ROS production in hypoxic breast cancer cells. MRPL52 promoted hypoxia adaptation and apoptotic resistance of BC cells by facilitating mitophagy to delay the onset of the vicious cycle between mitochondrial damage and ROS generation under hypoxia; At the same time, MRPL52 mediated hypoxia-induced ROS generation within a window, which further activated the ROS-Notch1-Snail signaling pathway to mediate the EMT and metastasis of hypoxic BC cells. Collectively, our work elucidated the intrinsic mechanism of MRPL52 upregulation in BC cells to sense decreased O_2_ levels that in turn allow survival and progression of tumors. We found that MRPL52 might mediate the influence of a heterogeneous tumor microenvironment on cancer cell behaviours and could be a promising therapeutic target for effective treatment of metastatic BC.

## Methods

### Clinical tissue samples and ethics statement

The experimental protocol was approved by the research ethics committee of the First Affiliated Hospital of China Medical University (approval number: 2019-72-2) and in strict accordance with the Declaration of Helsinki. Fresh BC tissues and matched adjacent normal tissues (ANTs) were obtained from BC patients undergoing modified radical mastectomy at the Department of Breast Surgery in the First Affiliated Hospital of China Medical University (Shenyang, China). The basic information of the included individuals is listed in Table S1. All participants or their guardians signed a written informed consent prior to the study. The patients had not received pre-surgical chemotherapy or radiotherapy and all resected tissues were pathologically confirmed. All samples were collected immediately following surgery, placed in liquid nitrogen overnight, and stored at -80°C before further examinations.

### Cell culture and transfection

Human BC cell lines (MCF-7, MDA-MB-231, BT-474), a normal breast epithelial cell line (MCF-10A) and a mouse BC cell line (4T1) were obtained from ATCC (Manassas, USA). The human BC cell lines T-47D was purchased from the cell bank of the Shanghai Institutes for Biological Sciences of the Chinese Academy of Sciences (Shanghai, China). MCF-7, MDA-MB-231, BT-474, and T-47D cells were cultured in high-glucose Dulbecco's modified Eagle's medium (DMEM; HyClone) with 10% fetal bovine serum (FBS; Gibco), 1% penicillin and 100 μg/mL streptomycin. 4T1 cells were cultured in RPMI 1640 medium (Invitrogen, Carlsbad, USA) with 10% FBS. MCF-10A cells were cultured in DMEM/F12 medium. All cells were stored in a humidified incubator at 37°C with 20% O_2_ and 5% CO_2_ (Thermo, Waltham, USA). The cells that needed hypoxic culture were moved to a hypoxic incubator chamber (37°C; 1% O_2_, 5% CO_2_; Thermo Scientific, USA) when they reached 70% to 80% confluence.

MDA-MB-231 and MCF-7 cells were seeded in six-well plates (Corning, NY, USA) and transiently transfected with small interfering RNAs (Si-RNAs) targeting MRPL52, HIF-1α, HIF-2α, Snail, PINK1, Parkin or their negative control (Si-NC) plasmid and plasmids overexpressing MRPL52, HIF-1α, Snail, N1ICD or empty control plasmids (empty vector; GenePharma, Shanghai, China) using Lipofectamine 3000 reagents (Invitrogen) according to the product manuals. After 6 h of coculture with vectors, the medium was replaced. At 24 h post-transfection, the cells in the normoxic group were maintained in a 20% O_2_ incubator, while the cells in the hypoxic groups were moved into a hypoxic chamber for 24 h, followed by subsequent experimentation. Lentiviruses were constructed by co-transfecting the 293T cell line with the packaging plasmid Sh-MRPL52 (LV-Sh-MRPL52), MRPL52 (LV-MRPL52) or their negative control (LV-Sh-NC and LV-Vector) (GenePharma, Shanghai, China), and the lentiviruses were collected after 48 h and used to infect 4T1 cells. The stably transfected cells were selected by puromycin (2 μg/mL; Solarbio, Beijing, China) for selection.

### RNA isolation and quantitative real-time PCR (RT-qPCR)

Total RNA from tissues and cells was isolated using TRIzol reagent (Ambion, Berlin, Germany). With the PrimeScript^TM^ RT reagent Kit with gDNA Eraser (Takara Bio, Beijing, China), total RNA was reverse transcribed into complementary DNA (cDNA) for mRNA detection. A Light Cycler 480 II Real-Time PCR system (Roche Diagnostics, Basel, Switzerland) was used to identify gene expression levels with SYBR^®^ Green (Takara Bio). Relative mRNA levels were normalized to the level of GAPDH and calculated by the 2^-ΔΔCt^ method 65. The relevant primers used were all synthesized by Sangon Biotech (Shanghai, China), with sequences listed as follows: MRPL52 (Forward, 5′-TTCTCTTCAGTGTCCGGAG-3′; Reverse, 5′-TATGACCAGTCTGGGAGCT-3′); Snail (Forward, 5′-CGCGCTCTTTCCTCGTCAG-3′; Reverse, 5′-TCCCAGATGAGCATTGGCAG-3′); Slug (Forward, 5′-CCAAACTACAGCGAACTGGAC-3′; Reverse, 5′-AGCTGAGGATCTCTGGTTGTG-3′); ZEB-1 (Forward, 5′-AAGAATTCACAGTGGAGAGAAGCCA-3′; Reverse, 5′-CGTTTCTTGCAGTTTGGGCATT-3′); ZEB-2 (Forward, 5′-AAGTATGTACTGACATAACC-3′; Reverse, 5′-GCTCTAAAGGAAGCAATCAT-3′); mtRNR1 (Forward, 5′-TAGAGGAGCCTGTTCTGTAATCGAT-3′; Reverse, 5′-CGACCCTTAAGTTTCATAAGGGCTA-3′) and mtRNR2 (Forward, 5′-CGCCTGTTTATCAAAAACAT-3′; Reverse, 5′-CTCCGGTTTGAACTCAGATC-3′; the corresponding genes of mitochondrially encoded 12 S rRNA and 16 S rRNA, respectively).

### RNA-sequencing (RNA-seq)

Total RNA containing mRNA was extracted from BC tissues with > 3 lymph node metastasis, BC tissues without any metastasis and ANTs as described above. Then mRNA sequencing libraries were generated using the NEB Next Ultra RNA Library Prep Kit (Illumina). RNA-seq was performed on the Illumina Novasq6000 platform by CapitalBio Technology (Beijing, China). Basic analysis mainly included raw data filtered by RNA-seq data analysis method were mapped to the hg38 genome with HISAT2 (Johns Hopkins University, USA). For the analysis of gene expression, fragments per kilobase of transcript per million mapped reads (FPKM) and read counts of each unigene were calculated. Differentially expressed genes (DEGs) were identified using R software (version: 3.5.1) with the DESeq2 package (http://bioconductor.org/packages/release/bioc/html/DESeq2.html). A q-value of ≤ 0.05 in multiple tests and an absolute log2-fold change value > 2 was used as the threshold for determining significant differences in gene expression. The DEGs selected above were compared with Gene Ontology (GO) and pathway annotation.

### Western blotting (WB)

Total protein contents were extracted with a Total Protein Extraction Kit (KeyGen Biotech, Nanjing, China). For extraction of cytosolic and mitochondrial proteins, a Cytoplasmic and Mitochondrial Protein Extraction kit (Beyotime, China) was used. Nuclear protein was extracted using a Nuclear Protein Extraction kit (Beyotime, China). Proteins were separated by 10% SDS-polyacrylamide gel electrophoresis (PAGE), and electrophoretically transferred onto polyvinylidene fluoride (PVDF) membranes (Millipore, USA). The membranes were blocked with 5% skim milk in TBST and then incubated with primary and secondary antibodies [peroxidase-conjugated AffiniPure goat anti-rabbit or anti-mouse IgG (H+L); ZSGB-BIO, Beijing, China]. The membranes were finally imaged by GelCapture software (DNR Bio-Imaging Systems, Jerusalem, Israel). As internal controls for normalization, endogenous specific protein markers were used: anti-COX IV antibody (1:5000, Abcam), as a mitochondrial control, anti-histone H3 antibody (1:5000, Abcam), as a nuclear control, and anti-β-actin (1:5000, Abcam) and anti-GAPDH (1:1000, Cell Signaling Technology) antibodies for cytosolic or total protein extracts. The following primary antibodies were used: N-cadherin (1:1000), ZO-1 (1:1000) and E-cadherin (1:1000), which were purchased from Cell Signaling Technology. Cyt c (1:1000) antibodies were purchased from BD Biosciences. GPX4 (1:1000), Parkin (1:1000), PINK1 (1:600), ND2 (1:1000) and Snail (1:1000) antibodies were purchased from Proteintech. Vimentin (1:1000) and cleaved PARP (1:400) antibodies were purchased from Santa Cruz Biotechnology. HIF-1α (1:800), HIF-2α (1:1000), ND5 (1:1000), CYTB (1:1000), COX1 (1:8000), COX2 (1:1000), ATP5A (1:5000), NDUFA9 (1:2000), Notch1 (1:1000), N1ICD (1:500), p62 (1:250), LC3B (1:2000), cleaved Caspase-3 (1:500), MRPL52 (1:500), and Caspase-1 (1:1000) antibodies were purchased from Abcam.

### Cell migration and invasion assays

Transwell migration assays were carried out using Transwell chambers (Corning). MDA-MB-231 and MCF-7 cells (2.5 × 10^5^ cells) were plated in the upper chamber with 200 μL of FBS-free DMEM, and 600 μL of DMEM with 10% FBS was added to the lower chamber. After 24 h, cells remaining on the upper surfaces were wiped off, while cells on the subjacent surface were washed, fixed and stained with hematoxylin and eosin (H&E), and the quantity of stained cells was counted under a microscope (Leica, Germany). Transwell invasion assays were performed using Matrigel (BD Biosciences, USA) following a similar method as described above.

### Cell viability and proliferation analysis

The viability of the cells was measured by a cell counting kit-8 assay (CCK-8; Dojindo, Japan) every day for 5 days. 2×10^3^ MDA-MB-231 and MCF-7 cells were plated in 96-well plates. Cells in each well were incubated with 10 μL of CCK-8 reagent for 1 h at 37℃, and the absorbance at 450 nm was measured by a microplate reader (Bio-Rad, Hercules, CA, USA). For cell proliferation detection, EdU-positive cells were identified by an EdU Cell Proliferation Kit (Beyotime, China) according to the manufacturer's specifications.

### Colony formation assay

For colony formation assays, 1×10^3^ MDA-MB-231 or MCF-7 cells were plated in 6-well plates and transfected. After 14 days of incubation at 37 °C, the colonies were fixed with 4% paraformaldehyde and stained with crystal violet solution (Solarbio). The visible colonies were counted with a microscope.

### Cell apoptosis assay

Apoptosis in cells was measured by terminal deoxynucleotidyl transferase-mediated dUTP nick-end labelling (TUNEL) staining using a One Step TUNEL Apoptosis Assay Kit (Beyotime, China). Images of TUNEL- and DAPI-stained cells were captured by a fluorescence microscope. The percentage of TUNEL-positive nuclei (red+blue) in the total DAPI-stained nuclei (blue) was determined.

### Fluorescence colocalization analysis

Cells were seeded into 6-well plates and transfected with adenovirus-LC3-EGFP (HANBIO, Shanghai, China) to mark autophagosomes. After incubation overnight, the cells were cultured under 1% O_2_ conditions for 24 h. The cells were stained with MitoTracker Red CMXRos (Beyotime, China) to mark mitochondria. The cells were imaged using a confocal fluorescence microscope (Leica) and the colocalization number of LC3-EGFP with mitochondria was counted.

### Detection of cytosolic ROS (cROS) and mitochondrial ROS (mROS)

For the ROS assay, the fluorescence probe DCFH-DA (Beyotime, Beijing, China) and MitoSOX Red (Thermofisher, Shanghai, China) were used to measure cROS and mROS, respectively. MDA-MB-231 and MCF-7 BC cells were labelled with DCFH-DA at 37°C for 20 min or MitoSOX Red reagents at 37°C for 10 min, rinsed three times, imaged by fluorescence microscopy and calculated as the mean intensity.

### Detection of mitochondrial membrane potential (MMP, Δψ_m_) and mitochondrial permeability transition pore (mPTP) opening

MMP was measured by a MMP assay kit (JC-1; Beyotime, Beijing, China). MDA-MB-231 and MCF-7 cells were incubated with the fluorescent probe JC-1 at 37°C for 20 min, washed three times and imaged by fluorescence microscopy. The ratios of red to green fluorescence intensity were used to assess the MMP. The mPTP opening was detected by calcein AM staining using a MPTP Assay Kit (Beyotime, Beijing, China). MDA-MB-231 and MCF-7 cells were incubated with calcein AM working solution plus fluorescence quenching solution at 37°C for 40 min. Replace fresh medium and continue to incubate cells at 37°C for an additional 30 min. Cells were washed three times, imaged by fluorescence microscopy and calculated as the mean intensity.

### Subcellular localization of MRPL52

The MRPL52 overexpression vector was cloned into the pEx-GFP plasmid (GFP-MRPL52) and transfected into MDA-MB-231 BC cells. After 48 h, cells were washed with PBS, and stained with MitoTracker Red CMXRos (Beyotime, China) and DAPI at 37°C. The image was visualized using a confocal microscope (Leica).

### Chromatin immunoprecipitation (ChIP) assay

ChIP was conducted using a SimpleChIP Kit (Cell Signaling Technology, USA). MDA-MB-231 and MCF-7 cells were cross-linked with 1% formaldehyde, quenched in glycine, lysed in SDS buffer and digested. Sheared DNA was rotationally incubated with antibodies against HIF-1α (Abcam), HIF-1β (Cell Signaling Technology), or IgG (Abcam) overnight at 4℃. The antibody-protein-DNA complex was resuspended with ChIP-grade Protein G magnetic beads and rotationally incubated for 2 h at 4 ℃. The precipitate was washed with low-salt buffer, followed by high-salt buffer. DNA elution was carried out with 1X ChIP elution buffer. Eluted DNA was de-cross-linked with 5 M NaCl and Proteinase K overnight at 65 ℃. The DNA fragment was extracted by a DNA Extraction Kit (Axygen) and quantified by RT-qPCR.

### Luciferase reporter assay

For construction of the luciferase reporter vectors of MRPL52, wild-type (WT) and mutant type (Mut) MRPL52 HREs were inserted into firefly luciferase reporter vectors (GenePharma, Shanghai, China). The plasmid expressing Renilla luciferase was used as the internal reference. MDA-MB-231 and MCF-7 cells were co-transfected with luciferase reporter constructs and HIF-1α-overexpressing plasmids or empty vector. After incubation overnight, cells were exposed to 20% or 1% O_2_ for 24 h. The firefly and Renilla luciferase activities were detected with a Dual-luciferase^®^ reporter assay (Promega) and an Infinite M200 Pro microplate reader (Tecan).

### Immunohistochemistry (IHC)

IHC was performed on 4-µm-thick sections from paraffin-embedded primary BC tissues and matched ANTs. The sections were dried, deparaffinized, rehydrated, and then boiled in citrate buffer (pH 6.0) for antigen retrieval. Following washing in Tris-buffered saline, tissues were incubated with 3% H_2_O_2_ at room temperature for 15 min for endogenous peroxidase activity block. Then, the slices were incubated with anti-MRPL52 antibody (ab121366, Abcam) at 4°C overnight. Anti-rabbit secondary antibodies from immunohistochemistry staining kits (ZSGB-BIO, Beijing, China) was added to each sample for 30 min, and subsequently visualized using 3,3′-diaminobenzidine (DAB; ZSGB-BIO, Beijing, China). Hematoxylin was used as a counterstain for sections. IHC staining of MRPL52 was analyzed and quantified by two independent pathologists using a double-blind method. The tissue sections were evaluated using an immunoreactivity score (IRS) system based on the staining intensity and proportion. The staining intensity was graded as follows: negative = 0, mild = 1, moderate = 2, and strong = 3. The proportion of positively stained cells was scored as follows: 0 represented 0-5% positive cells, 1 represented 5-30% positive cells, 2 represented 30-60% positive cells, and 3 represented ≥ 60% positive cells. The IRS was represented by intensity multiplying proportion scores. For the degree of MRPL52 staining, IRS > 3 was defined as high expression and IRS ≤ 3 was defined as low expression.

### Immunofluorescence (IF)

IF staining was carried out in both tissue sections and cell samples seeded on coverslips within 24-well plates. The samples were fixed in 4% paraformaldehyde and permeabilized with 0.1% Triton X-100. The slides were then blocked for 1 h with 2% BSA, after which they were incubated with primary antibodies overnight at 4℃, and then with CY3-conjugated secondary antibodies (CWBIO, Beijing, China) for 1 h at room temperature. Finally, DAPI was used for nuclear staining. For the tumor hypoxia region analysis, 5 × 10^5^ 4T1 cells were injected orthotopically into the mammary fat pads of five female BALB/c mice (Charles River, Beijing, China). Three weeks later, the mice were intraperitoneal injected with pimonidazole (60 mg/kg; Hypoxyprobe, USA). After 2 hours, tumors were resected, fixed and cryosectioned. The cryosections were fixed with ice-cold methanol. To detect the pimonidazole, tumor sections were incubated with FITC-conjugated anti-pimonidazole mouse monoclonal antibody (Hypoxyprobe Green Kit). Sections were subsequently co-stained with MRPL52 for IF detection.

### Transmission electron microscopy (TEM)

MDA-MB-231 and MCF-7 cells plated on culture dishes were fixed with 4% glutaraldehyde for 1 h at 4 ℃, after which, the cells were scraped off, centrifuged at low speed, and stored in 1.5 mL of 4% glutaraldehyde at 4 ℃. Subsequently, glutaraldehyde was replaced with 0.2 M sucrose overnight. The cells were fixed with 1% osmium tetroxide for 1 h, rinsed and dehydrated with rising concentrations of ethanol, and then embedded in TAAB Epon (Marivac, Canada). The cells were sectioned 60 nm thick, collected on copper grids, and stained with uranyl acetate and lead citrate. Images were captured with a transmission electron microscope (Hitachi Ltd., Tokyo, Japan).

### Animal model

For the *in vivo* orthotopic xenograft implantation assay, female BALB/c mice (6 weeks old, weighing 16-20 g, acquired from Charles River, Beijing) were randomly divided into 4 groups (n = 5 per group); the MRPL52 overexpressed group, the MRPL52 knockdown group and the control groups. The stably-transfected 4T1 cells were validated by RT-qPCR, suspended in 100 μL of PBS plus 100 μL of Matrigel substrate and injected into the 4^th^ coupled mammary fat pad of mice at a density of 5 × 10^5^ cells. Tumor volumes were measured every 3 days using the following formula: length × width^2^ × 0.5 (cm^3^). Mice were sacrificed on the 24^th^ day post-injection, and tumors were removed and weighed. The volume of the tumor was calculated and a tumor growth curve was then plotted. Then the xenografts were imaged, fixed in 4% paraformaldehyde, and embedded in paraffin. The green fluorescein-based TUNEL assay of the tissues was performed by an *In Situ* Cell Death Detection Kit (Roche, Germany). The lungs and livers of mice were collected, fixed in 4% paraformaldehyde, embedded in paraffin and stained by hematoxylin and eosin (H&E). The animal experimental procedures were approved by the China Medical University Institutional Ethics Committee and followed the Guide for the Care and Use of Laboratory Animals (US National Institutes of Health publication, Doc. 2011-11490).

### Statistical analyses

All data analyses were performed using GraphPad Prism software (version 8.0), with results expressed as the mean ± standard deviation. Chi-Square test and Pearson's χ^2^ test were used to analyze patient data. Student's t test was used to compare two groups, while one way analysis of variance (ANOVA) was used for multiple data groups. All experiments were repeated in least triplicate. A P value < 0.05 was considered statistically significant.

## Results

### MRPL52 upregulation is negatively related to the clinical outcomes of human BC

**Figure 1:**
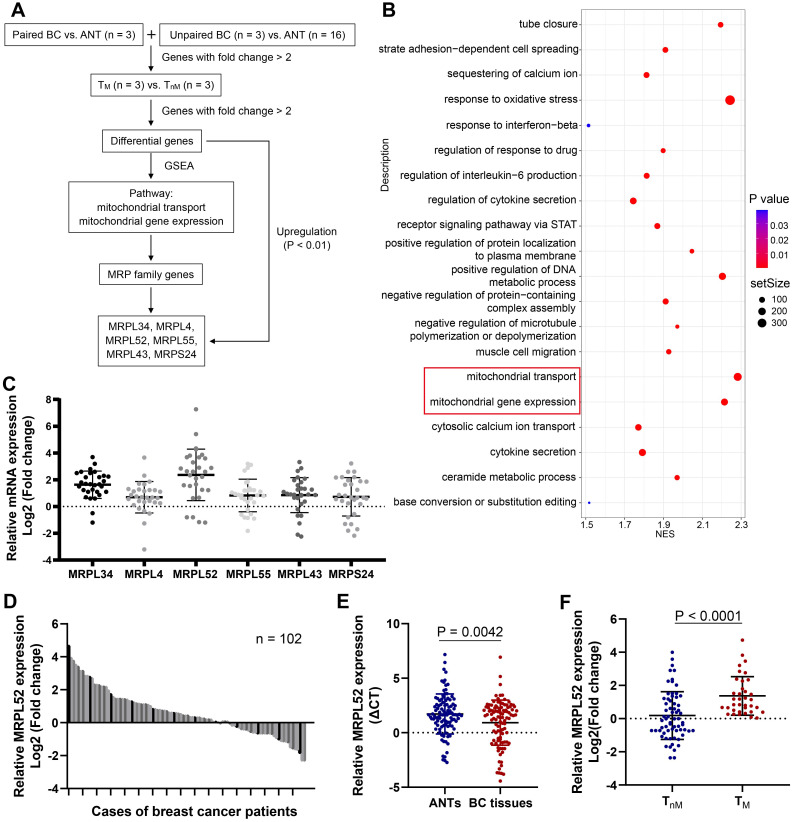
MRPL52 is upregulated in human BC. (A) Screening rationale of the RNA-seq assay. The screened genes were upregulated by more than 2-fold in breast cancer (BC) tissues and BC tissues with > 3 lymph nodes metastasis (T_M_) compared with adjacent normal tissues (ANTs) and BC tissues without lymph node metastasis (T_nM_), respectively. (B)The Bubble Chart of the results of GSEA was generated using the R package 'ggplot2'. Red represents a P value < 0.05 and redder colors indicate lower P values. (C) RT-qPCR of MRPL34, MRPL4, MRPL52, MRPL55, MRPL43 and MRPS24 expression in 30 human BC tissues and their paired ANTs. Data are shown as mean ± SD. (D) RT-qPCR of MRPL52 expression in 102 human BC tissues and their paired ANTs. Data are shown as mean ± SD. (E) MRPL52 expression in unpaired BC tissues and ANTs from clinical samples. Higher Ct value indicated lower gene expression. Data are shown as mean ± SD. (F) MRPL52 expression in T_nM_ and T_M_ tissues from clinical samples. Data are shown as mean ± SD.

**Figure 2:**
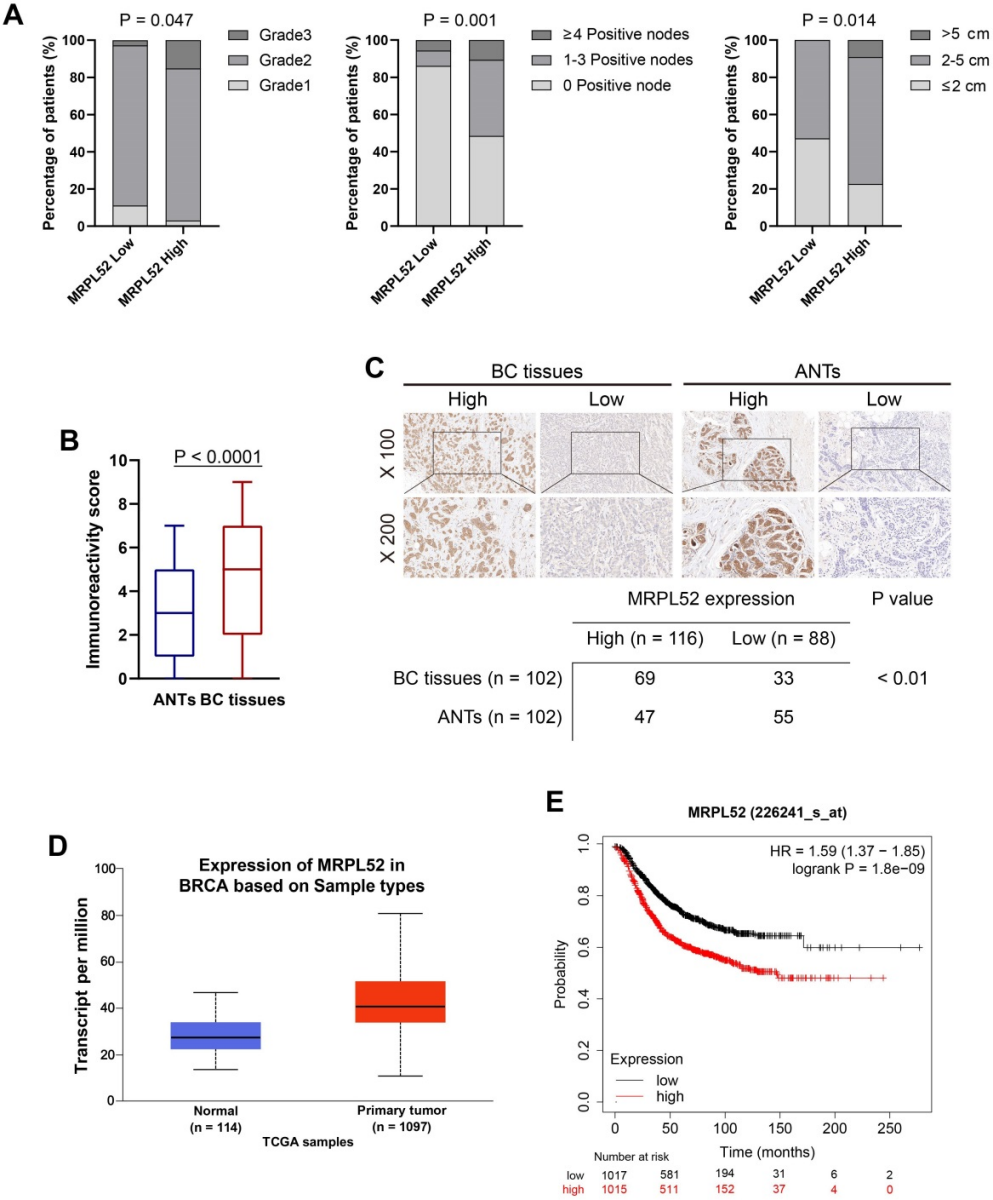
MRPL52 upregulation is negatively related to the clinical outcomes of human BC. (A) Chi-Square test of correlations between MRPL52 expression level and clinicopathological characteristics of our clinical BC patients in order to confirm the potential clinical value of MRPL52. (B) The different immunoreactivity scores of MRPL52 in BC tissues and ANTs detected by IHC staining. (C) Upper panel, the representative IHC images of MRPL52 protein expression in human BC tissues and ANTs. Lower panel, expression of MRPL52 in BC tissue and ANTs by IHC. Statistical analysis was carried out by Pearson's χ^2^ test. (D) Data of MRPL52 expression in patients with BC from the database UALCAN. (E) Kaplan-Meier plot of RFS based on MRPL52 expression in BC downloaded from the Kaplan-Meier plotter database.

To screen for significant genes in BC that are potentially associated with BC metastasis, we performed RNA-seq assays of our clinical BC samples, and the screening rationale is shown in Figure 1A. Gene Set Enrichment Analysis (GSEA) was performed on differential genes between BC tissues from metastatic (T_M_) and non-metastatic (T_nM_) patient cohorts. The pathways with a significant enrichment (P < 0.05) are listed in Figure 1B, among which two mitochondria-related pathways (mitochondrial transport and mitochondrial gene expression) draw our attention. Interestingly, we noted that the MRP family genes account for more than half of total genes enriched in the two pathways. Therefore, six MRP family candidate genes that were enriched and upregulated in RNA-seq (P < 0.01) have been screened out. The expression of these genes were further verified in clinical samples, which demonstrated that MRPL52 exhibited the highest expression in BC tissues compared with ANTs (Figure 1C). As indicated, the correlated genes of MRPL52 were enriched in GO terms closely related to mitochondria, including mitochondrial gene translation, ETC complex assembly and electron transfer activity (Figure S1A-B). Consistent with the transcriptome sequencing results, MRPL52 was differentially overexpressed in our clinical samples from 102 BC patients (Figure 1D-E). The T_M_ displayed higher MRPL52 expression than T_nM_ in a 5-year follow-up period (Figure 1F). MRPL52 overexpression was strongly correlated with more advanced clinicopathological features, including histological grade, lymph node metastasis and tumor size (Figure 2A and Table S1). Additionally, IHC was carried out on BC tissues and matched ANTs to evaluate the protein level of MRPL52, which validated the high expression of MRPL52 in BC tissues compared with ANTs (Figure 2B-C). The upregulation of MRPL52 in BC (P < 0.001) was further verified through the analysis tool UALCAN (Figure 2D) 66. High mRNA expression of MRPL52 in BC was significantly associated with a poorer recurrence-free survival (RFS) [hazard risk (HR) = 1.59, 95% confidence interval (CI): 1.37-1.85, P = 1.8E-09] (Figure 2E). Collectively, these findings revealed that MRPL52 is a potential prognostic marker gene in human BC, suggesting that upregulation of MRPL52 may play a key role in BC progression.

### MRPL52 acts as a novel transcriptional target of HIF-1 in response to hypoxia

**Figure 3:**
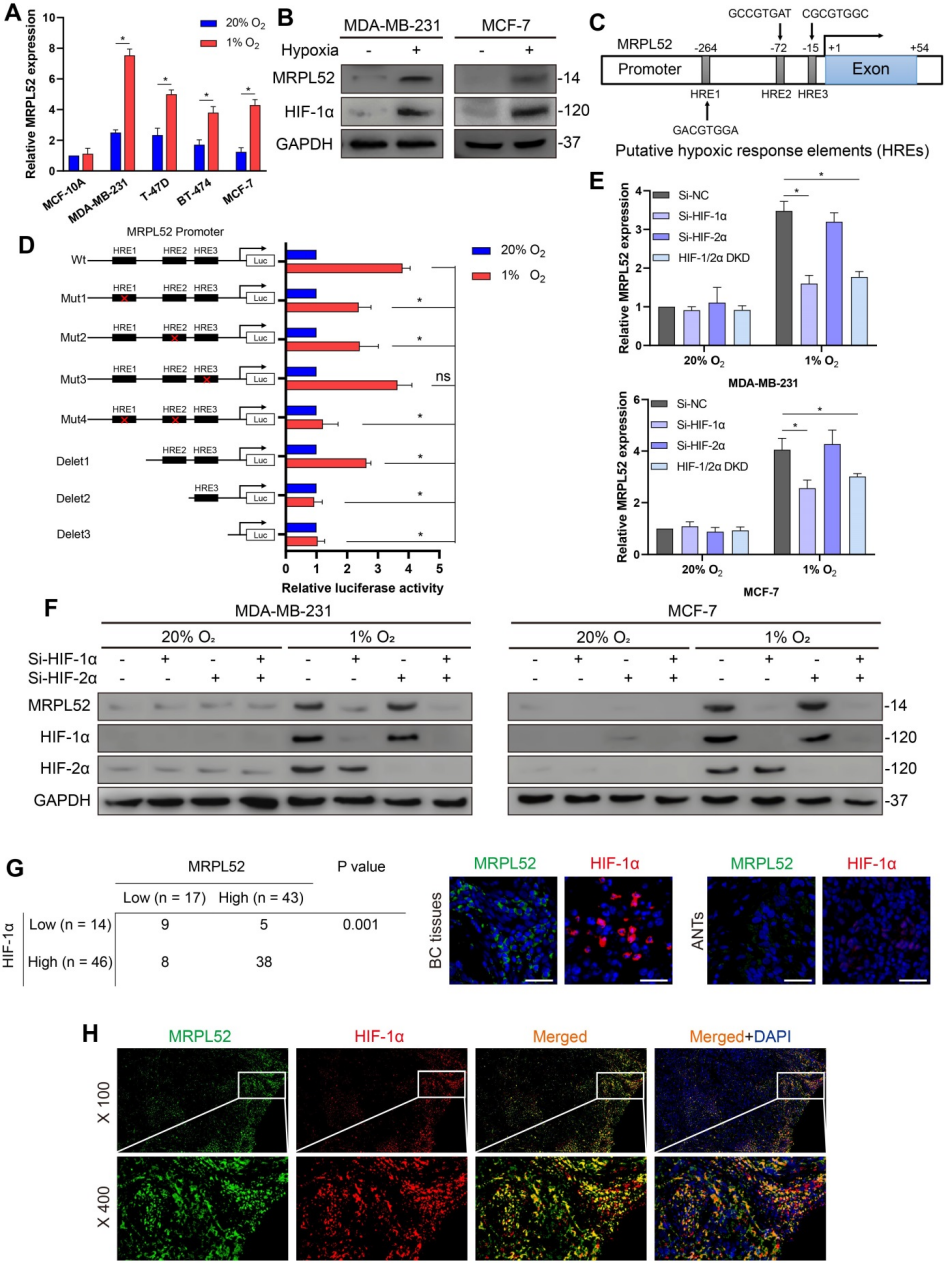
MRPL52 is transcriptionally activated by HIF-1 in response to hypoxia. (A) RT-qPCR analysis of MRPL52 mRNA levels in 4 BC cell lines and 1 normal breast epithelial cell line exposed to 20% or 1% O_2_ (mean ± SD, n = 4). *P < 0.05. (B) WB analysis of MRPL52 and HIF-1α in MDA-MB-231 and MCF-7 cell lines exposed to 20% or 1% O_2_. (C) The JASPAR website was used to predict the potential hypoxic response elements (HREs) in the promoter of MRPL52. (D) Luciferase activities of MRPL52 promoter with the wild type and the mutated HREs in MDA-MB-231 cells exposed to 20% or 1% O_2_ were determined using a dual-luciferase reporter assay (mean ± SD, n = 3). *P < 0.05. Wt, wild type; Mut, mutant type; Delet, serial deletion; HREs, hypoxic response elements; Luc, luciferase reporter plasmid. (E) RT-qPCR and (F) WB assays were performed to analyze MRPL52 expression level in BC cell under normoxia or hypoxia (mean ± SD, n = 4). *P < 0.05. (G) Left panel, expression of MRPL52 and HIF-1α in 60 BC patients detected by tissue IF. Statistical analysis was carried out by Pearson's χ^2^ test. Right panel, the representative IF images of MRPL52 (green) and HIF-1α (red) expression in human BC tissues and matched ANTs. Scale bars, 25µm. (H) Representative IF images of MRPL52 (green) and HIF-1α (red) in human BC tissues. Yellow color in the merged image represents the co-localization of MRPL52 and HIF-1α.

We investigated whether the high expression of MRPL52 in BC is partially caused by the hypoxic tumor environment. We performed RT-qPCR and WB in 4 human BC cell lines that were exposed to 1% or 20% O_2_ for 24 h, and noted that the mRNA and protein level of MRPL52 was consistently and significantly enhanced by hypoxia (Figure 3A-B, Figure S1C). Then, we analysed the sequence of human MRPL52 gene promoter and found 3 candidate sites for HREs (5'-CGTG-3') at -264 bp, -72 bp and -15 bp (HRE1, HRE2 and HRE3, respectively; Figure 3C). The results of serial deletion and site-directed mutagenesis suggested that HRE1 and HRE2 are involved in hypoxia-induced transactivation of MRPL52 (Figure 3D). To determine whether HIFs are essential transcription factors for activating the HREs of MRPL52 promoter, we have constructed Si-RNA targeting HIFs. Figure 3E-F revealed that HIF-1α knockdown and HIF-1/2α double knockdown (DKD), but not HIF-2α knockdown, reversed hypoxia-induced MRPL52 mRNA and protein upregulation in BC cells. IF double staining of MRPL52 and HIF-1α was carried out in 60 paired samples of human BC tissues. Among the tumors with higher expression of HIF-1α (46 of 60), 38 (82.6%) were positive for MRPL52, however, only 5 (35.7%) of the tumors negative for HIF-1α (14 of 60) highly expressed MRPL52 (Figure 3G). Additionally, the IF staining assay also showed the co-localization of MRPL52 and HIF-1α expression in BC tissue specimens (Figure 3H). Notably, ectopic expression of HIF-1α profoundly enhanced the luciferase activity of MRPL52 promoter in MDA-MB-231 and MCF-7 BC cells exposed to 20% or 1% O_2_ (Figure S1D). ChIP assays further showed apparent fold enrichment of HIF-1α and HIF-1β on the putative sites HRE1 and HRE2 of MRPL52 promoter compared to the IgG control in BC cells exposed to 1% O_2_ (Figure S1E). All these evidence demonstrates that MRPL52 is upregulated in BC cells and tissues exposed to hypoxia and acts as a transcriptional target of HIF-1 in response to hypoxia.

### MRPL52 supports the survival of BC cells exposed to hypoxia

**Figure 4:**
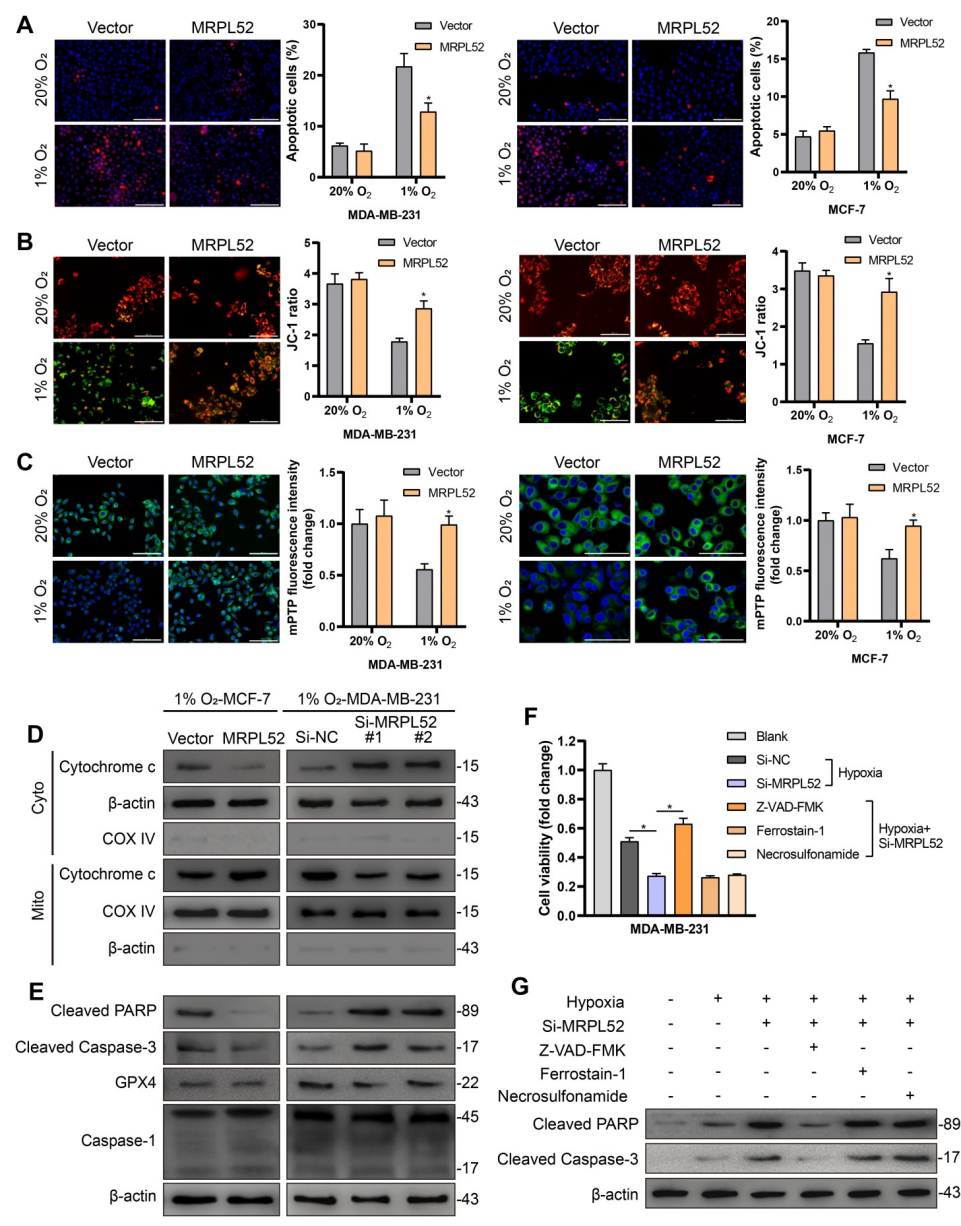
MRPL52 resists the caspase-dependent mitochondrial apoptosis of BC cells exposed to hypoxia. (A) Cellular apoptosis examined by TUNEL staining (mean ± SD, n = 3). *P < 0.05. Scale bars, 100 µm. (B) MMP detected by JC-1 probe. JC-1 red (JC-1 aggregate) shows healthy MMP, whereas JC-1 green (JC-1 monomer) shows decreased MMP. ΔΨ_m_ was represented as the red/green ratio (mean ± SD, n = 3). *P < 0.05. Scale bars, 100 µm. (C) Alterations in mitochondrial permeability transition pore opening were detected by calcein AM staining. The weaker the green fluorescence in cells, the higher the degree of opening of mPTP (mean ± SD, n = 3). *P < 0.05. Scale bars, 100 µm. The proteins expression of (D) Cytochrome c in cytoplasm and mitochondria, (E) cleaved PARP, cleaved Caspase-3, GPX4 and Caspase-1 were analyzed using WB. Cyto, cytosolic protein; Mito, mitochondrial protein. (F-G) MDA-MB-231 cells were incubated with Z-VAD-FMK (50 μM), a pan-caspase inhibitor or ferrostatin-1 (20 nM), a ferroptosis inhibitor or necrosulfonamide (20 nM), a necroptosis inhibitor for 24 h. (F) Cell viability, (G) cleaved PARP and cleaved Caspase-3 levels were measured using CCK-8 and WB analysis, respectively (mean ± SD, n = 3). *P < 0.05.

To study the role of MRPL52 in the survival of BC cells exposed to low oxygen, we performed gain- and loss-of-function studies in MDA-MB-231 and MCF-7 cell lines. The transfection efficacy is shown in Figure S2A. CCK-8 assays showed that MRPL52 overexpression enhanced the viability of BC cells exposed to 20% or 1% O_2_, whereas knocking down MRPL52 under hypoxia led to decreased numbers of living cells (Figure S2B). This result was further confirmed by colony formation assay. The colony counts were enhanced by MRPL52 upregulation in BC cells exposed to 20% or 1% O_2_ (Figure S2C), whereas MRPL52 knockdown under hypoxia caused a reduction in colony number (Figure S2D). EdU assays indicated that MRPL52 slightly enhanced the proliferative capacity of BC cells under normoxic conditions, but not under hypoxic conditions (Figure S2E-F). The results of TUNEL staining and JC-1 assays were combined to demonstrate that MRPL52 overexpression inhibited (Figure 4A-B), whereas silencing MRPL52 promoted (Figure S2G-H) the mitochondrial apoptotic pathway in hypoxic BC cells. However, under normoxia, MRPL52 led to a negligible effect on BC cell apoptosis (Figure 4A-B). Moreover, MRPL52 upregulation reduced, while MRPL52 downregulation promoted the extent of mPTP opening induced by hypoxia, but this effect was not found in normoxic BC cells (Figure 4C, Figure S3A).

We next performed a WB assay to confirm the molecular mechanism required for MRPL52 to resist apoptosis in hypoxic BC cells. The results showed that MRPL52 upregulation reduced, whereas MRPL52 knockdown promoted Cyt c release from mitochondria into cytosol induced by hypoxia in BC cells (Figure 4D). The protein levels of cleaved Caspase-3 and cleaved PARP in hypoxic BC cells increased with MRPL52 downregulation and decreased with MRPL52 overexpression; however, GPX4 (ferroptosis-related index) and Caspase-1 (pyroptosis-related index) did not show changes in expression (Figure 4E). Subsequently, hypoxic MDA-MB-231 cells with MRPL52 knockdown were treated with three different apoptotic inhibitors, whose cell viability was estimated by CCK-8 assays. The results showed that only Z-VAD-FMK (pancaspase inhibitor) reversed the effect of MRPL52 knockdown in hypoxic BC cells (Figure 4F). Consistently, cleaved Caspase-3 and cleaved PARP levels were reduced by Z-VAD-FMK in MDA-MB-231-Si-MRPL52 cells exposed to 1% O_2_ (Figure 4G). Collectively, our data suggest that MRPL52 acts as a caspase-dependent mitochondrial apoptotic pathway repressor rather than a proliferating activator in BC cells under hypoxia.

### MRPL52 is involved in hypoxia-induced BC cell EMT, migration and invasion

**Figure 5:**
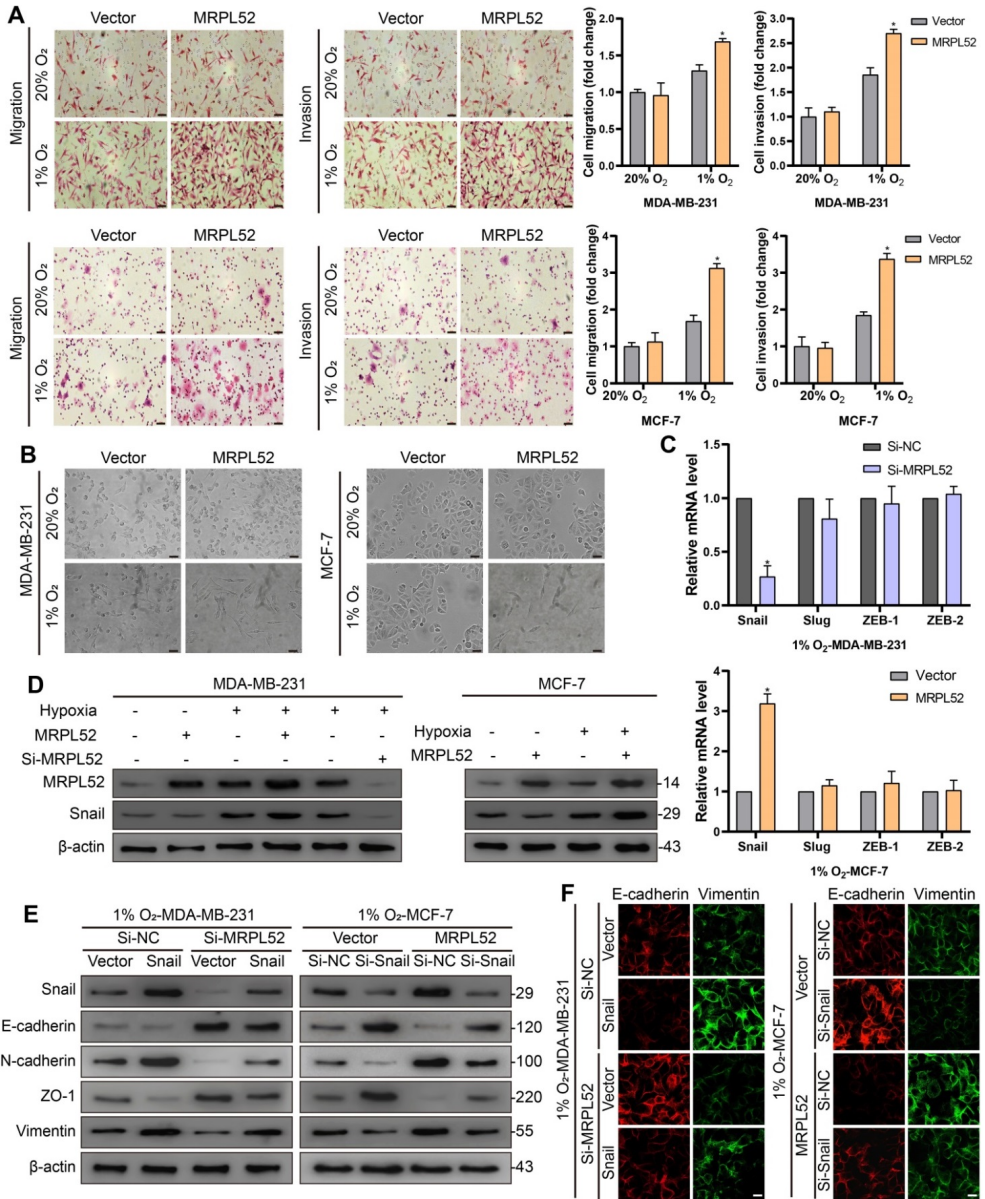
MRPL52 mediates hypoxia-induced EMT, migration and invasion of BC cell. (A) Ability of cell migration and invasion tested by Transwell assay (mean ± SD, n = 3). *P < 0.05. Scale bars, 50 µm. (B) The morphological changes of cells undergoing EMT include the acquisition of spindle-liked phenotype and the loss of polarity and cell-cell adhesion, which are observed by an optical microscope. Scale bars, 50 µm. (C) The expression levels of Snail, Slug, ZEB-1 and ZEB-2 under hypoxia assessed by RT-qPCR (mean ± SD, n = 3). *P < 0.05. (D) The protein expression of MRPL52 and Snail was evaluated by WB. (E) WB and (F) IF were performed to assess the protein expression levels of Snail, E-cadherin, N-cadherin, ZO-1 and Vimentin. Scale bars, 50 µm.

Transwell assays showed that overexpression of MRPL52 promoted (Figure 5A), whereas downregulation of MRPL52 suppressed (Figure S3B) the migration and invasion of BC cells under hypoxia. Since EMT is a master mechanism that initiates the process of tumor migration and invasion, we further observed the role of MRPL52 in EMT. Hypoxia treatment induced an apparent epithelial-to-mesenchymal morphological conversion in MDA-MB-231 and MCF-7 cells, the effect of which was abrogated by MRPL52 knockdown and enhanced by MRPL52 upregulation (Figure 5B and Figure S3C). In addition, four critical EMT-associated transcriptional regulators were screened by RT-qPCR, among which, Snail was the only one whose mRNA level was significantly increased by MRPL52 overexpression and decreased by MRPL52 knockdown in BC cells exposed to 1% O_2_ (Figure 5C). The effect of MRPL52 on the protein level of Snail in hypoxic BC cells was further validated (Figure 5D). As indicated, Si-MRPL52-induced inhibition of cell migration and invasion was reversed by Snail overexpression (Figure S3D), while the promoting effect of MRPL52 overexpression on metastasis was blocked by Snail silence (Figure S3E). Next, WB and IF assays were applied to confirm that MRPL52 increased the expression of N-cadherin, and Vimentin (mesenchymal markers), and decreased the expression of E-cadherin and ZO-1 (epithelial markers) via provoking Snail expression in hypoxic BC cells (Figure 5E-F). These results suggested that MRPL52 plays an essential role in initiating hypoxia-induced EMT, migration and invasion of BC cells.

### *In vivo* effects of MRPL52 on breast cancer growth and metastasis

**Figure 6:**
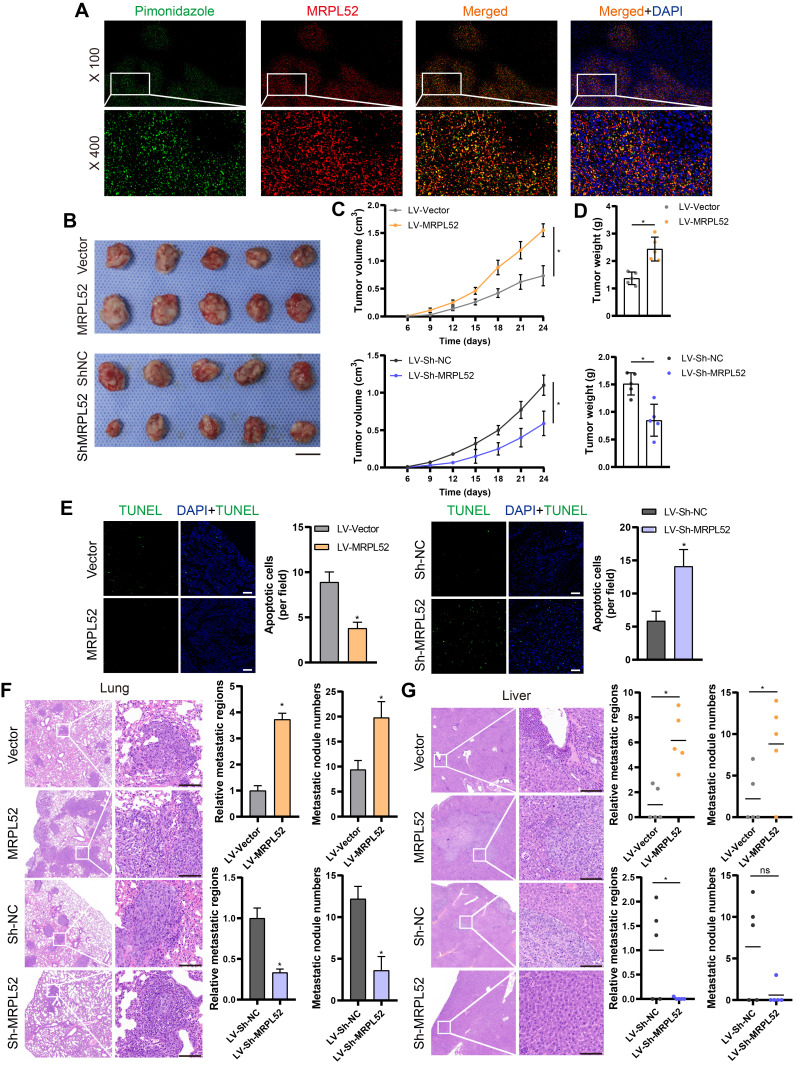
MRPL52 promotes the survival and metastasis of BC cells *in vivo*. (A) Representative IF images of MRPL52 (red) and pimonidazole (green) in 4T1 xenograft tumors. Yellow color in the merged image represents co-localization of MRPL52 and pimonidazole. (B) Images, (C) growth curves and (D) weights of xenograft tumors (mean ± SD, n = 5). *P < 0.05. Scale bars, 1 cm. (E) TUNEL staining of xenografts (mean ± SD, n = 5). *P < 0.05. Scale bars, 100 µm. H&E analysis of (F) lung and (G) liver metastasis in mice. Scale bars, 100 µm. The relative metastatic regions (tumor area/organ area) were calculated according to H&E analysis (mean ± SD, n = 5). The microscopically visible metastatic nodules on the surface of lung and liver were counted (mean, n = 5). *P < 0.05.

In order to validate the endogenous upregulation of MRPL52 induced by hypoxia in solid tumors, we used pimonidazole, a hypoxia marker, to identify hypoxic regions in 4T1 xenograft tumors. Results of IF staining showed that MRPL52 and pimonidazole were co-localized in xenografts (Figure 6A). Stable MRPL52-overexpressing cell lines (LV-MRPL52), stable MRPL52-knockdown cell lines (LV-Sh-MRPL52) and their corresponding control cell lines (LV-Vector and LV-Sh-NC) were established by lentivirus-mediated plasmids delivery in 4T1 cells (Figure S3F). We performed an orthotopic xenograft implantation assay in female BALB/c mice whose mammary fat pads were injected with stably transfected 4T1 cells. As a result, the primary tumors formed by LV-MRPL52 cells were larger than that formed by LV-Vector cells, while tumors with MRPL52 knockdown were smaller than tumors in LV-Sh-NC group (Figure 6B-D). Knockdown of MRPL52 increased, while overexpressing MRPL52 reduced the ratio of apoptotic (TUNEL-positive) cells in xenografts, as detected by an *in situ* cell death detection kit (Figure 6E). Figure 6F-G indicated that MRPL52 upregulation significantly enhanced 4T1 cells metastasis to the lung and liver, whereas, MRPL52 knockdown drove an opposite effect. Based on these results, the roles of MRPL52 in apoptotic resistance and metastatic promotion of BC were further validated *in vivo*.

### MRPL52 reduces apoptosis of hypoxic BC cells by promoting the clearance of damaged mitochondria

**Figure 7:**
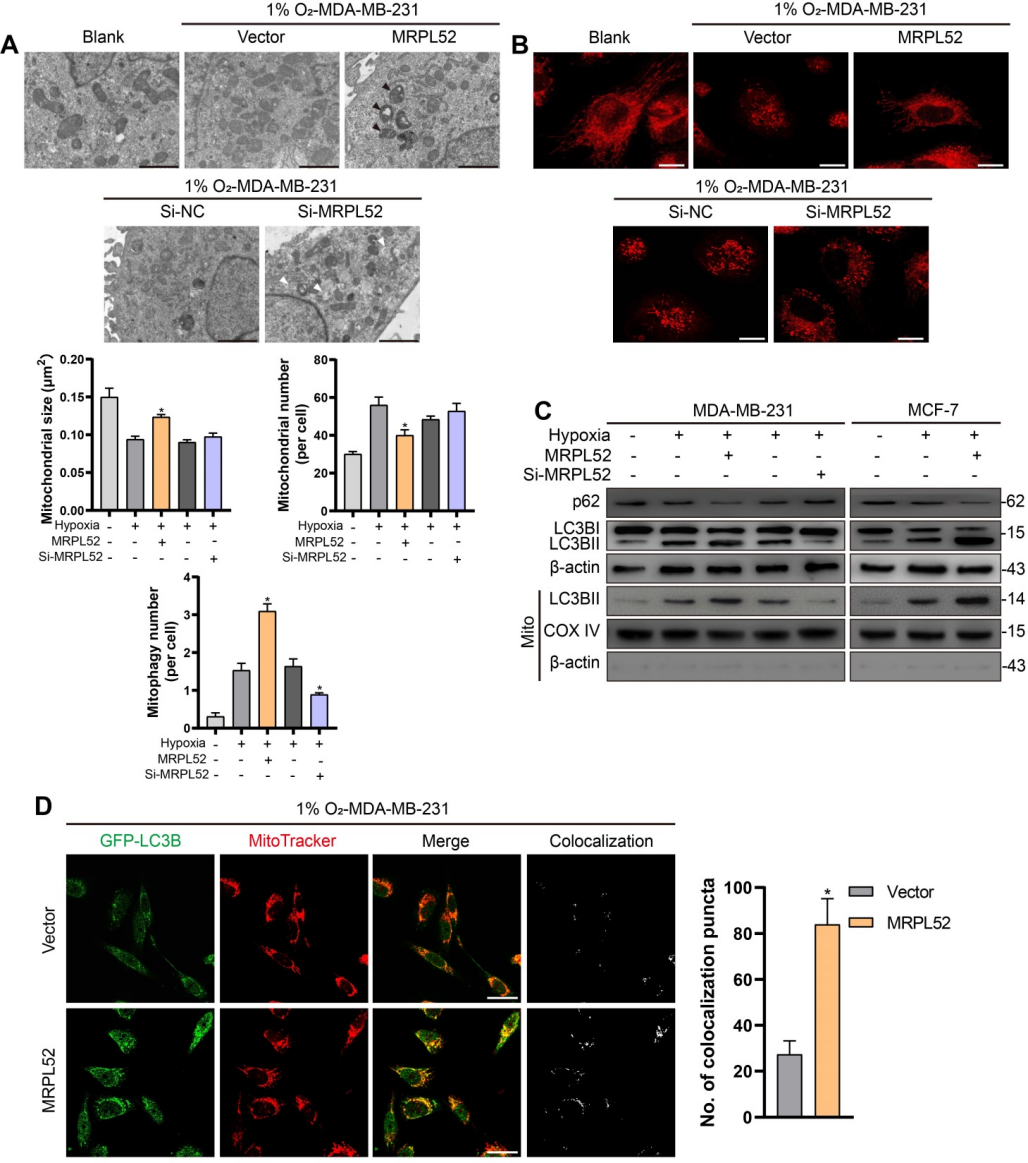
MRPL52 promotes mitophagy in hypoxic BC cells. (A) Mitochondrial ultrastructure in MDA-MB-231 and MCF-7 cells under TEM. Black arrows point to mitophagy and white arrows point to vacuolar deformation of mitochondria. Scale bars, 2 µm. (B) Mitochondria were stained using MitoTracker probe. Scale bars, 5 µm. (C) Autophagy induction was investigated by examining the protein levels of p62 and LC3B by WB. The LC3BII protein level on mitochondrial fraction was assessed by WB in order to detect the mitophagy. Mito, mitochondrial protein. (D) MDA-MB-231 cells were co-transfected with GFP-LC3B and the indicated plasmid, cultured under hypoxia for 24 h, and stained with MitoTracker probe. The colocalization of LC3B puncta (green) with mitochondria puncta (red) marked by yellow fluorescent intensity was analyzed by confocal fluorescence microscopy (mean ± SD, n = 3). *P < 0.05. Scale bars, 10 µm.

Since the mitochondrial pathway has been found to be one of the main regulators of apoptosis, we further observed the effects of MRPL52 on mitochondria in hypoxic BC cells. TEM was performed to study the effects of MRPL52 on mitochondrial morphology in BC cells (Figure 7A and Figure S4A). The results demonstrated that BC cells exposed to 20% O_2_ showed intact and clear mitochondrial cristae. Under hypoxic conditions, the cristae remained intact, whereas the increased mitochondria number and decreased mitochondria size in BC cells might be attributed to mitochondrial fission in response to hypoxia. Mitochondrial fission is a process in which damaged parts of mitochondria are separated to become dysfunctional small-sized mitochondria. However, when MRPL52 was overexpressed, increased mitophagy reactions and a reduced number of abnormal mitochondria were observed. In contrast, the structure of mitochondria transformed from layered into dilated, which was characterized by vacuolation and incomplete cristae in 231-Si-MRPL52 cells under hypoxia. These results were further validated by MitoTracker staining, in which knocking down MRPL52 increased the number of damaged mitochondria whose morphology changed from a long-spindle into a small-dotted phenotype, whereas MRPL52 overexpression reduced small-sized mitochondria in hypoxic BC cells (Figure 7B). WB revealed that MRPL52 overexpression led to increased LC3B-II/I ratio and decreased p62 expression in hypoxic BC cells, while MRPL52 knockdown played an opposite role (Figure 7C). The recruitment of LC3B-II to the mitochondrial fraction was greater in BC cells exposed to 1% O_2_ than in BC cells exposed to 20% O_2_ and could be further enhanced by MRPL52 upregulation and inhibited by MRPL52 knockdown (Figure 7C). Moreover, MRPL52 overexpression elevated the number of colocalization puncta formed by autophagosomes and mitochondria in hypoxic MDA-MB-231 BC cells (Figure 7D).

**Figure 8:**
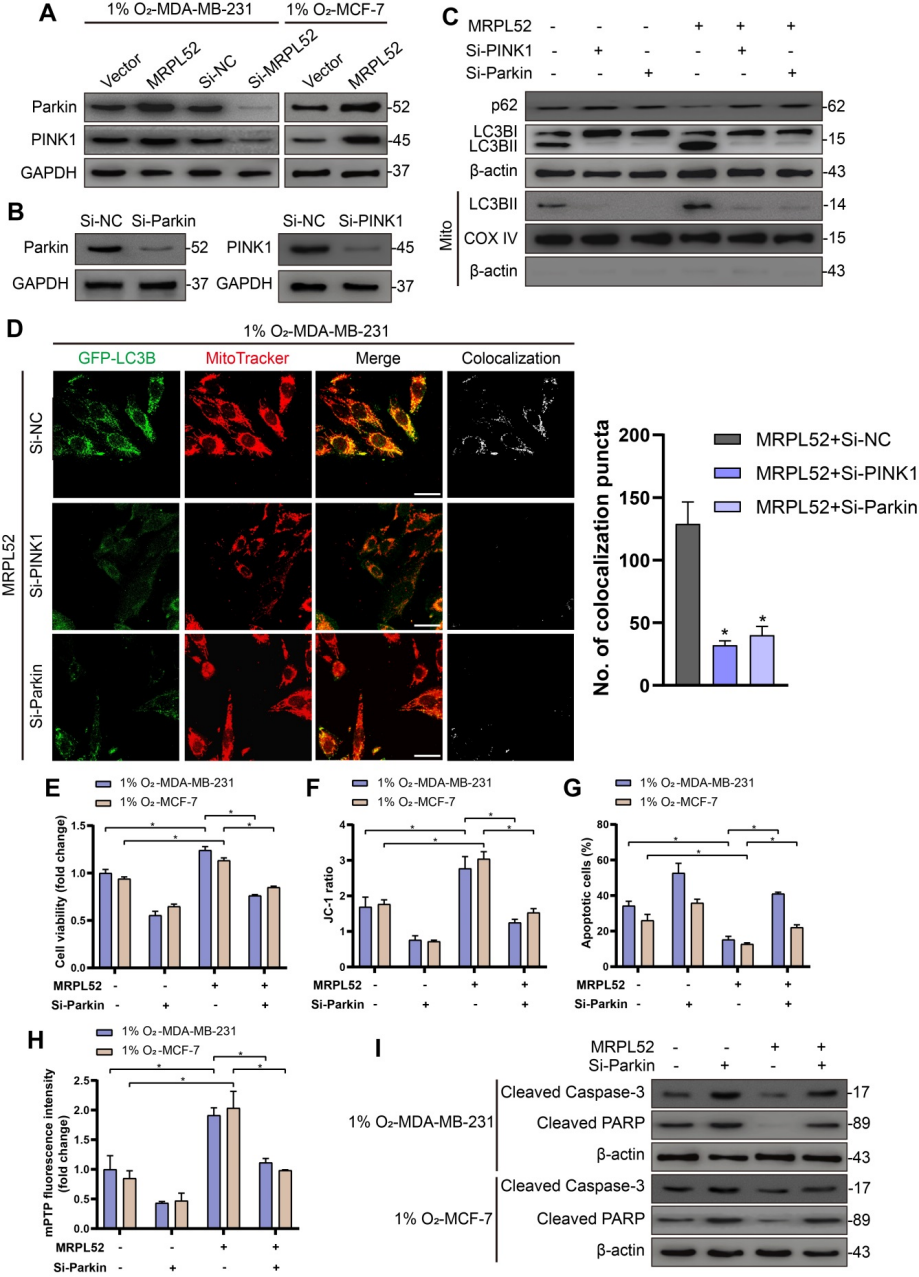
MRPL52 reduces apoptosis of hypoxic breast BC cells by PINK1/Parkin-mediated mitophagy. (A) Protein levels of Parkin and PINK1 assessed by WB. (B) Transfection efficacy evaluated by WB. (C) Autophagy induction was investigated by examining the protein levels of p62 and LC3B by WB. The LC3BII protein level on mitochondrial fraction was assessed by WB in order to detect the mitophagy. Mito, mitochondrial protein. (D) MDA-MB-231 cells were co-transfected with GFP-LC3B and the indicated plasmid, cultured under hypoxia for 24 h, and stained with MitoTracker probe. The colocalization of LC3B puncta (green) with mitochondria puncta (red) marked by yellow fluorescent intensity was analyzed by confocal fluorescence microscopy (mean ± SD, n = 3). *P < 0.05. Scale bars, 10 µm. (E) Cell viability assessed by CCK8 assay (mean ± SD, n = 4). *P < 0.05. (F) Mitochondrial membrane potential detected by JC-1 probe (mean ± SD, n = 3). *P < 0.05. (G) Cellular apoptosis examined by TUNEL staining. (mean ± SD, n = 3). *P < 0.05. (H) Alterations in mitochondrial permeability transition pore opening were detected by calcein AM staining. The weaker the green fluorescence in cells, the higher the degree of opening of mPTP (mean ± SD, n = 3). *P < 0.05. (I) The protein expressions of cleaved PARP and cleaved Caspase-3 were analyzed using WB.

In addition, we observed that the protein levels of PINK1 and Parkin in hypoxic BC cells were elevated by MRPL52 overexpression and decreased by MRPL52 downregulation (Figure 8A). To confirm that MRPL52-mediated promotion of mitophagy in hypoxic BC cells is mediated by the PINK1/Parkin pathway, we transfected BC cells with PINK1-SiRNA and Parkin-SiRNA (Figure 8B). The results of WB and fluorescence colocalization analysis revealed that knockdown of PINK1 or Parkin differentially decreased the colocalization of LC3 and mitochondria in MRPL52 overexpressed MDA-MB-231 cells under hypoxia (Figure 8C-D). Therefore, MRPL52 is responsible for mitophagy through the PINK1/Parkin pathway in BC cells, a process that plays a protective role in cellular adaptation to hypoxic microenvironment. Conversely, knocking down MRPL52 promoted the accumulation of damaged mitochondria with an abnormal structure and function. Subsequently, we observed that cell viability and Δψ_m_ were decreased and that cell apoptosis and extent of mPTP opening was enhanced in 1% O_2_-MDA-MB-231-MRPL52 and 1% O_2_-MCF-7-MRPL52 BC cells transfected with Parkin-SiRNA compared to those transfected with Si-NC (Figure 8E-H). The MRPL52-induced decreases in protein levels of cleaved Caspase-3 and cleaved PARP were further abrogated by Parkin knockdown (Figure 8I). Our data showed that MRPL52 is responsible for suppressing cellular apoptosis of hypoxic BC cells by promoting mitophagy to remove destroyed mitochondria quickly, rescue the Δψ_m_ and reduce Cyto C release into cytoplasm.

### MRPL52 mediates EMT in hypoxic BC cells through the activation of ROS-Notch1-Snail signaling pathway

**Figure 9:**
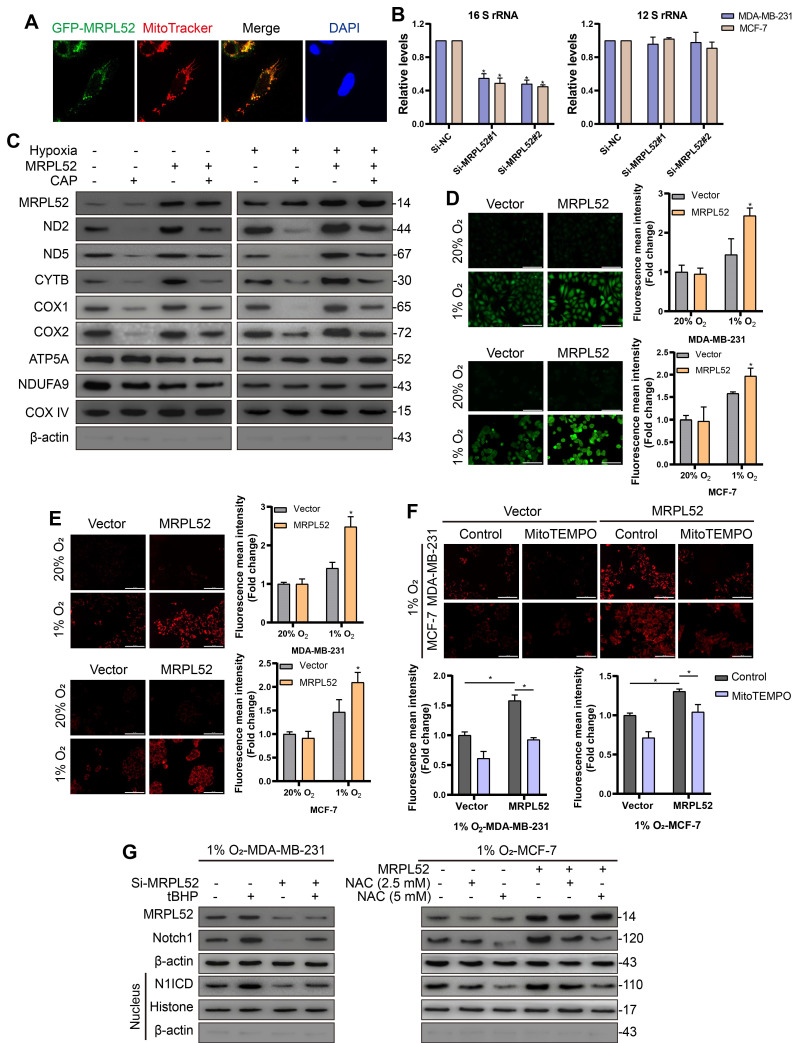
MRPL52 activates the ROS-Notch1-Snail signaling pathway to mediate EMT of BC cells exposed to hypoxia. (A) MDA-MB-231 cell was transfected with GFP-tagged MRPL52 in order to analyze the subcellular localization of MRPL52. (B) Levels of mitochondrial 12 S and 16 S rRNA were evaluated by RT-qPCR (mean ± SD, n = 4). *P < 0.05. (C) Mitochondrial proteins were isolated and treated by CAP (0.15 μg/μL) for 10 min at 4 ℃ as required. WB was performed to assess protein levels of mitochondrial respiratory complexes components, including ND2 and ND5 (complex I), CYTB (complex III), COX1 and COX2 (complex IV) encoded by mt-DNA, as well as ATP5A and NDUFA9 encoded by nuclear genome. (D) DCFH-DA and (E) MitoSOX fluorescence in cells measured by fluorescence microscopy. The fluorescence mean intensity of DCFH-DA and MitoSOX represent cytosolic ROS and mitochondrial ROS, respectively (mean ± SD, n = 3). *P < 0.05. Scale bars, 100 µm. (F) Cells were preincubated with MitoTEMPO (20 μM) for 1 h. MitoSOX fluorescence in cells measured by fluorescence microscopy (mean ± SD, n = 3). *P < 0.05. Scale bars, 100 µm. (G) Cells were preincubated with tBHP (200 μM) for 4 h; or with NAC (2.5 or 5 mM) for 1 h. WB for detecting the expression levels of MRPL52, Notch1 and N1ICD in MDA-MB-231 and MCF-7 cells exposed to 1% O_2_ with treatments as indicated.

As expected, the localization of GFP-MRPL52 signals completely overlapped with the red fluorescence of the mitochondrion-specific dye MitoTracker (Figure 9A), suggesting that MRPL52 protein is localized in the mitochondria. Mitoribosomes consist of a small subunit that is assembled by 12 S rRNA and MRPS and a large subunit that includes 16 S rRNA and MRPL. We assessed the levels of mt-12 S rRNA and mt-16 S rRNA to reflect the assembly of small or large subunits. The level of 16 S rRNA was decreased, whereas the 12 S rRNA level remained unchanged when MRPL52 was knocked down in MDA-MB-231 and MCF-7 cells (Figure 9B). Thus, MRPL52 is responsible for the assembly of the large subunit of mitoribosomes. Then, we carried out assays to determine the role of MRPL52 in mtDNA expression. As shown in Figure 9C, overexpressing MRPL52 increased the protein levels of several mitochondrial respiratory complex components in BC cells exposed to 20% or 1% O_2_, the effects of which were further abrogated by the mitochondria-specific translation inhibitor, chloramphenicol (CAP), whereas the nuclear-encoded mitochondrial proteins NDUFA9 and ATP5A did not vary. Therefore, MRPL52 facilitates ETC complex protein synthesis by promoting the translation of mtDNA. Since CYTB is a central subunit of ETC complex III, through which mROS released to intermembrane space (IMS) is increased under hypoxia 55, 67, 68, we postulated that MRPL52 could affect the ROS generation in hypoxic BC cells. As indicated, MRPL52 upregulation apparently increased both cROS and mROS levels in hypoxic BC cells, but not in BC cells exposed to 20% O_2_ (Figure 9D-E); And Si-MRPL52 dramatically abolished the elevated ROS generation in BC cells caused by hypoxic exposure (Figure S4B-C). Under hypoxia, MRPL52 upregulation-mediated promotion of mROS production was abolished by MitoTEMPO, a mitochondrially targeted antioxidant that specially scavenges mitochondrial superoxide (Figure 9F). Taken together, MRPL52 mediates the hypoxia-induced increase in ROS production in BC cells.

Notch family members serve as receptors for membrane-bound ligands and are involved in tumorigenesis. Importantly, Notch signaling has been reported to mediate hypoxia-induced EMT of cancer cells by directly increasing the expression of Snail or Slug 69-71. And Notch1 has been reported to be activated by an increased level of intracellular ROS 72. Notch1 signaling has been found to be profoundly activated in human BC, which correlates with higher invasiveness and poorer prognosis 73-75; therefore, we observed the role of ROS and Notch1 in MRPL52-mediated EMT and metastasis of hypoxic BC cells. As shown by WB, MRPL52 knockdown differentially reduced the expression levels of Notch1 in the cytoplasm and its active form, the Notch1 intracellular domain (N1ICD), in the nucleus, which could be reversed by tert-butylhydroperoxide (tBHP), a ROS generation agent. Conversely, MRPL52 overexpression promoted Notch1 and N1ICD expression, which was abolished by the nonspecific ROS scavenger N-acetylcysteine (NAC) (Figure 9G). Furthermore, tBHP treatment and N1ICD overexpression rescued the inhibitory effect of Si-MRPL52 on EMT (Figure S4D-E). Transwell assays also showed that tBHP and N1ICD blocked the MRPL52 knockdown-mediated suppression of hypoxic BC cell migration and invasion (Figure S5A, C). In contrast, the treatment with NAC and the Notch signaling inhibitor DAPT abrogated the mesenchymal transition (Figure S4D-E) and decreased metastatic capacity (Figure S5B and Figure S6A) induced by MRPL52 overexpression. Collectively, MRPL52 mediates migration and invasion of BC via promoting ROS production to activate the ROS-Notch1-Snail-EMT pathway in BC cells exposed to hypoxia.

Taken together, our study reveals the critical role of hypoxia-induced gene MRPL52 in the adaptation and progression of BC in response to hypoxic microenvironment by maintaining moderate production of ROS and relieving the accumulation of oxidatively damaged mitochondria.

## Discussion

Our study identified MRPL52, a component of the mitoribosome large subunit, as a transcriptional target of HIF-1 in BC cell lines exposed to hypoxic conditions. The significance of our study is manifold. First, we confirmed that MRPL52 was significantly upregulated in human BC, which led to poorer prognosis and aggressive clinicopathological features. Second, MRPL52 was shown to be vital for overcoming the hypoxia-triggered vicious cycle between ROS and mitochondria in BC cells by promoting PINK1/Parkin-mediated mitophagy to eliminate oxidatively damaged mitochondria. Thus, the inhibitory effect of MRPL52 on caspase-dependent mitochondrial apoptosis of the BC cells exposed to 1% O_2_ was potently confirmed in our experiments. Third, further experiments demonstrated that MRPL52 promotes a moderate increase in ROS generation which augments hypoxic BC cell EMT, migration, invasion and metastasis through the activation of the ROS-Notch1-Snail signaling pathway. The moderate increase in ROS generation maybe a result of the bidirectional regulatory effect of MRPL52 on ROS production. Therefore, this study strikingly suggests that a hypoxia-responsive gene, MRPL52, serves as a key regulator of hypoxia-induced BC metastatic events and a mechanism required for hypoxic adaptation of BC cells. Some other MRPs have shown abnormal expression in BC, such as MRPL15 36, MRPL33 37 and MRPL54 38. However, the evidence for determining whether the conclusions in this paper are applicable to other MRPs is still limited at present, which is worth further exploration in the future.

Mitoribosome is composed of elements encoded from both the mitochondrial genome (the RNA components) and the nuclear genome (the MRPs). Mitochondrial genome-encoded circRNAs (mt-circRNAs) account for a considerable fraction of RNA components in the mitochondria, however, the biological function of mt-circRNAs is still not understood. Based on existing studies on the mt-circRNAs, steatohepatitis-associated circRNA ATP5B regulator (SCAR) plays a pivotal role in mROS metabolism and fibroblast activation, and mc-COX2 modulates mitochondrial function and regulates chronic lymphocytic leukemia cell proliferation and apoptosis 76, 77. Unlike the traditional circRNAs, the mt-circRNAs may work via new mechanisms instead of acting as the competing endogenous RNA. Future researches can further investigate whether mitochondrial RNAs may regulate the function of MRPs to affect mitochondria activity and cancer development.

The hypoxic microenvironment of BC is usually attributed to the rapid proliferation of tumors that outgrow the oxygen-supplying capacity of vascularization. As a common tumor microenvironmental factor, hypoxia plays a paradoxical role in cancer cells. Cellular organelle dysfunction and aberrant energy metabolism caused by oxidative stress may lead to irreversible damage to cancer cells. However, cancer cells under hypoxia exhibited various responses including enhanced angiogenesis 78, metabolic reprogramming 79, 80, and altered survival and metastasis 6, 8. A certain number of tumor cells may undergo apoptosis in hypoxic conditions, while the cancer cells that have survived under hypoxia probably possess more resistance to apoptosis and higher aggressive phenotype. A study demonstrated that post-hypoxic tumor cells have a survival advantage in the bloodstream and increased probability of overt metastasis 38. Thus, hypoxia and the signaling molecules linking hypoxia and malignant progression could represent promising targets for cancer therapy. HIFs are heterodimers consisting of two subunits, HIF-α and HIF-β. HIF-α is hydroxylated by prolyl hydroxylases and then ubiquitination degraded in the presence of O_2_. However, in the absence of O_2_, PHDs are inhibited by increased intracellular ROS; thus, HIF-α is stably expressed and forms a transcriptional complex with the constitutively expressed subunit HIF-β 81. HIF-1 and HIF-2 are the major factors driving adaptive reactions, such as metabolic reprogramming and angiogenesis in cancer cells in response to hypoxia 80, 82, 83. Previous studies have confirmed that HIF-1 played either promoting or inhibiting role in hypoxia-induced apoptosis. In our study, MRPL52 was identified as a cancer-promoting oncogene which mediated the apoptotic resistance and metastatic initiation of hypoxic BC cells. And the upregulation of MRPL52 in BC was found to be induced by HIF-1 under the hypoxic microenvironment. Therefore, MRPL52 can be considered as a molecular mechanism that mediates the hypoxia-induced malignant phenotype of tumor as well as the adaptation of cancer cell to hypoxia.

Mitophagy plays an important role in regulating mitochondrial number and maintaining mitochondrial function by removing dysfunctional and damaged mitochondria in cells. The PINK1/Parkin pathway acts as a principal mechanism for mitophagy, the activation of which depends on mitochondrial membrane depolarization 84. Enhanced mitophagy has been found to protect cancer cells from damage resulting from various causes, such as hypoxia, impaired electron transport and cytotoxic chemotherapy 85-87. Conversely, accumulated damaged mitochondria that suffer excessive oxidative stress show Δψ_m_ decreases, mitochondrial permeability transition (MPT) pore opening and mitochondrial depolarization, which lead to Cyt c release to cytosol, and trigger Caspase-3 activation and cell apoptosis. As shown in our study, MRPL52 upregulation could promote protective mitophagy in BC cells under hypoxic conditions; moreover, Parkin knockdown significantly abrogated MRPL52-mediated apoptotic suppression in hypoxic BC cells. Therefore, MRPL52 may prevent the accumulation of damaged mitochondria and uncontrolled generation of ROS by promoting PINK1/Parkin-mediated mitophagy. When MRPL52 was overexpressed in hypoxic breast cancer cells, it regulated the moderate generation of ROS which tended to activate signaling pathway, rather than start the vicious circle and trigger an apoptotic cascade. In contrast, knockdown of MRPL52 in hypoxic breast cancer cells slowed the clearance of damaged mitochondria. As a result, breast cancer cells exhibited lower threshold tolerance to ROS, in which the over-accumulation of damaged mitochondria initiated the caspases activation and the subsequent apoptotic program. Further studies aimed to elucidate the underlying mechanisms involved in MRPL52-promoted mitophagy in BC cells exposed to hypoxia.

It was reported that mitochondria with rich and abundant functions could support the proliferation and energy metabolism of cancer cells 20, 88. Nevertheless, mitochondrial dysfunction might be associated with tumor progression 89, 90. The results of our study reconcile the seemingly paradoxical role of mitochondria in cancer and illustrate the dynamic, variable and complex function of mitochondria in tumorigenesis. Under normoxia, the increased mitochondrial translation tends to optimize metabolic ability rather than cause a more incomplete reduction in molecular oxygen. Thus, MRPL52 upregulation did not lead to an apparent increase in ROS production. In addition, we identified the promoting effect of MRPL52 on BC cell proliferation under normoxia. Considering that MRPL33 protein can be detected in intracellular areas other than mitochondria, it is unclear whether the role of MRPL52 in BC proliferation depends on mitochondrial function alteration. In contrast, the upregulation of MRPL52 in hypoxic BC cells led to an increase in ROS production by facilitating the formation of ETC complex III. Indeed, the ETC complex III is the only complex in the respiratory chain that mediates increased mROS release under hypoxic conditions 67, 91. The outer ubiquinone binding site (Qo) of complex III may be a possible source of ROS, in which a low O_2_ level could prolong the lifetime of the semiquinone radical. The underlying mechanism still needs further exploration 92. ROS were once thought to be a factor that merely threatens the survival of cancer cells, and growing evidence has suggested that ROS can act as a signal transduction messenger in the oxygen sensing response to regulate hypoxic adaptation, tumor proliferation, and cell fate determination. The functions of mitochondria in tumorigenesis are not static but are altered depending on the tumor microenvironment and genetic discrepancies between tumors. The pluripotency of mitochondrial function endows BC cells with the flexibility to adapt and survive under different environmental conditions or stresses.

During EMT, epithelial cells lose polarity and cell-cell adhesion and acquire a mesenchymal phenotype, including spindle-shaped morphology and mesenchymal biomarker expression. EMT is regulated by multiple signaling pathways and acts as a critical step in initiating the metastatic cascade of cancer cells. Snail (Snail1) and Slug (Snail2) are two reliable transcription factors that suppress the expression of E-cadherin to initiate EMT in cancer, and both have been reported to be transcriptionally regulated by Notch signaling 69, 71, 93. The Notch family consists of four transmembrane receptors (Notch1-4) that are regulated by five ligands. The activation of Notch signaling leads to cleavage of the Notch receptor and release of the Notch intracellular domain (NICD) into nucleus to regulate the expression of various tumorigenesis-related targeted genes. In addition, J Chen et al. reported that Notch signaling is potentiated in BC cells exposed to hypoxia via HIF transcription factors 70. The increased level of intracellular ROS has been reported to upregulate Notch1 by inducing the release of nuclear factor erythroid-2 related factor 2 (Nrf2) from its repressor protein Kelch-like ECH-associated proteins (Keap1) 72. Here, we provide the evidence that MRPL52-mediated ROS production in response to hypoxia may be an initiating factor leading to the upregulation of Notch1, which increases the expression of Snail, thus promoting EMT, migration and invasion of hypoxic BC cells.

## Conclusions

In summary, we elucidated the functions and regulatory mechanisms of a novel HIF-1 target gene MRPL52 in BC cell adaptation, survival and metastasis under hypoxia. MRPL52 maintains hypoxic BC cell viability by promoting PINK1/Parkin-dependent mitophagy and mediates hypoxic-induced metastatic initiation of BC cells by transactivating the ROS-Notch1-Snail pathway. These findings identify MRPL52 as a potential prognostic biomarker as well as a possible therapeutic target for metastatic BC. Importantly, our study provides a better understanding of the mechanisms that allow alterations in mitochondrial function, structure and ETC composition in BC cells that evolved to adapt to the hypoxic microenvironment.

## Supplementary Material

Supplementary figures and tables.

## Abbreviations

IARC: International Agency for Research on Cancer
MRPL52: mitochondrial ribosome protein L52
ROS: reactive oxygen species
BC: breast cancer
HREs: hypoxia response elements
HIFs: hypoxia inducible factors
EMT: epithelial-mesenchymal transition
mtDNA: mitochondrial gene
ETC: electron transport chain
OXPHOS: oxidative phosphorylation
ANTs: adjacent normal tissues
Cyt c: Cytochrome c
Δψ_m_: mitochondrial membrane potential
MPT: mitochondrial permeability transition
DMEM: Dulbecco's modified Eagle's medium
CCK-8: cell counting kit-8 assay
TUNEL: terminal deoxynucleotidyl transferase-mediated dUTP nick-end labelling
IF: immunofluorescence
TEM: transmission electron microscopy
GSEA: Gene set enrichment analysis
RFS: recurrence-free survival
ChIP: chromatin immunoprecipitation
DKD: double knockdown
N1ICD: Notch1 intracellular domain
tBHP: tert-butylhydroperoxide
NAC: N-acetylcysteine
Keap1: Kelch-like ECH-associated proteins
CYTB: Cytochrome b
GPX4: Glutathione peroxidase 4
ND2: NADH dehydrogenase subunit 2
ND5: NADH dehydrogenase subunit 5
COX1: Cyclooxygenase 1
COX2: Cyclooxygenase 2
