# Supplementary material for: HIF-1-induced mitochondrial ribosome protein L52: a mechanism for breast cancer cellular adaptation and metastatic initiation in response to hypoxia
Source: Theranostics. 2021 May 25;11(15):7337–59. doi: 10.7150/thno.57804 (PMC8210597; doi:10.7150/thno.57804)
Supplement: Supplementary file 1 — Supplementary figures and tables. [file thnov11p7337s1.pdf]

Table S1 Clinicopathological characteristics of breast cancer patients

Variables	Cases (n = 102)	MRPL52 High No. (%)	MRPL52 Low No. (%)	P value
Age				
≤ 50	38	24 (63.2)	14 (36.8)	0.801
> 50	64	42 (65.6)	22 (34.4)	
Tumour size				
≤ 2 cm	32	15 (46.9)	17 (53.1)	
2-5 cm	64	45 (70.3)	19 (29.7)	0.014*
> 5 cm	6	6 (100.0)	0 (0)	
Molecular subtype				
Luminal A	27	22 (81.5)	5 (18.5)	
Luminal B	52	30 (57.7)	22 (42.3)	0.138
$HER2^+$	13	9 (69.2)	4 (30.8)	
TNBC	10	5 (50.0)	5 (50.0)	
Positive lymph nodes				
0	63	32 (50.8)	31 (49.2)	
1-3	30	27 (90.0)	3 (10.0)	0.001*
≥ 4	9	7 (77.8)	2 (22.2)	
Grade				
1	6	2 (33.3)	4 (66.7)	
2	85	54 (63.5)	31 (36.5)	0.047*a
3	11	10 (90.9)	1 (9.1)	
ER				
Negative	23	13 (56.5)	10 (43.5)	0.351
Positive	79	53 (67.1)	26 (32.9)	
PR				
Negative	28	16 (57.1)	12 (42.9)	0.326
Positive	74	50 (67.6)	24 (32.4)	

a, Using Fisher's exact test; *$P < 0.05$, statistically significant.

HER2, human epidermal growth factor receptor 2; TNBC, triple-negative breast cancer; ER, estrogen receptor; PR, progesterone receptor.

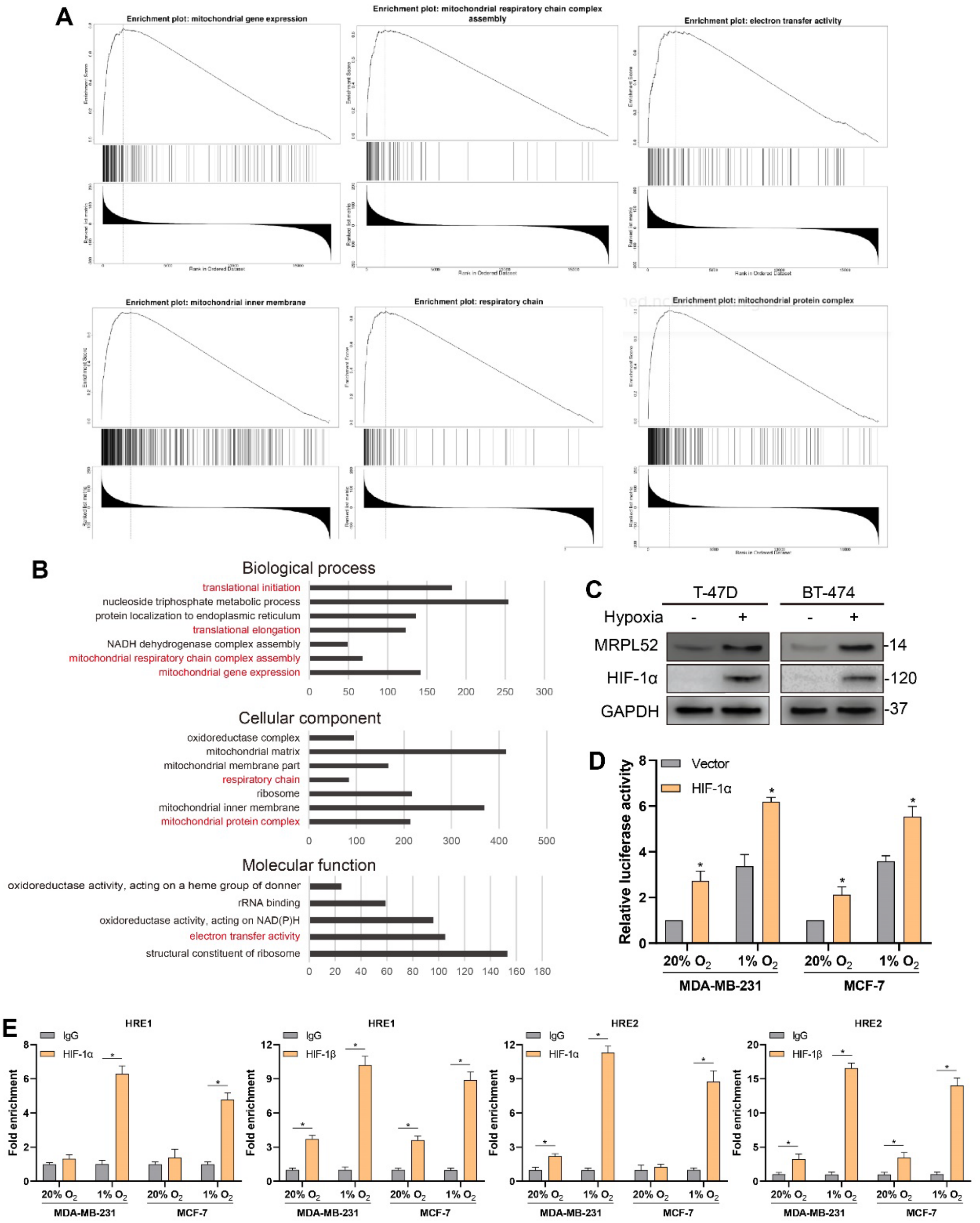

Figure S1 (A) GSEA performed using GSEA software. (B) Analysis of GO terms enrichment. GO, Gene Ontology. (C) WB analysis of MRPL52 and HIF-1α in T-47D and BT-474 cell lines exposed to 20% or 1% O_2. (D) Luciferase reporter assay of MRPL52 in MDA-MB-231 and MCF-7 cells exposed to 20% or 1% O_2 (mean ± SD, n = 3). *P < 0.05. (E) Validation of putative HIF-1-binding motifs in MRPL52 promoter using ChIP assay (mean ± SD, n = 4). *P < 0.05. HREs, hypoxic response elements.

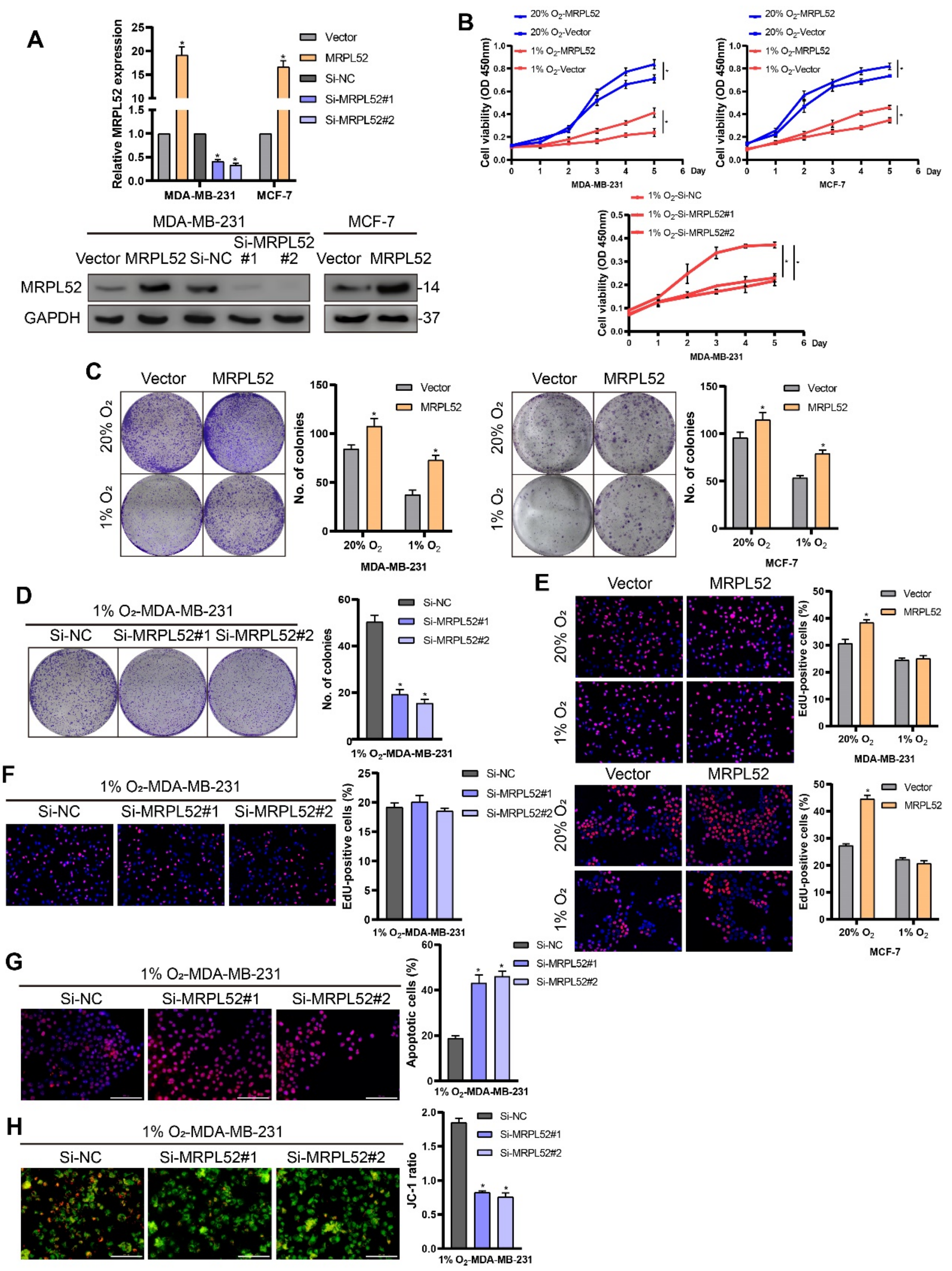

Figure S2 (A) The transfection efficacy detected by RT-qPCR and WB (mean ± SD, n = 4). *P < 0.05. (B) Cell viability assessed by CCK8 assay (mean ± SD, n = 3). *P < 0.05. (C-D) The colony formation assay of cells and the quantitative analysis of colonies (mean ± SD, n = 3). *P < 0.05. (E-F) EdU staining of cells and EdU-positive cell proportion was calculated (mean ± SD, n = 3). *P < 0.05. (G) Cellular apoptosis examined by TUNEL staining (mean ± SD, n = 3). *P < 0.05. Scale bars, 100 µm. (H) MMP detected by JC-1 probe (mean ± SD, n = 3). *P < 0.05. Scale bars, 100 µm.

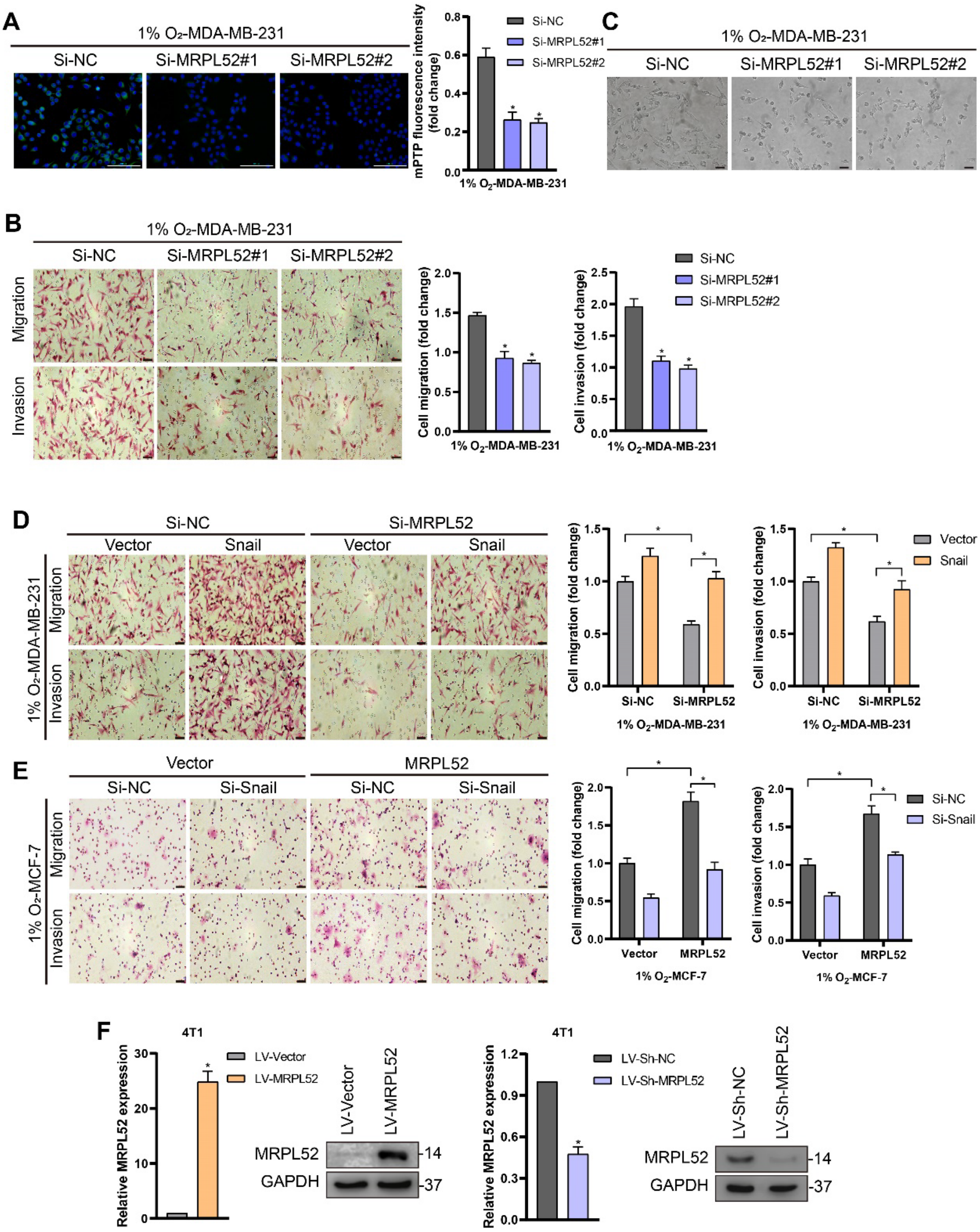

Figure S3 (A) Alterations in mitochondrial permeability transition pore opening were detected by calcein AM staining. The weaker the green fluorescence in cells, the higher the degree of opening of mPTP (mean ± SD, n = 3). *P < 0.05. Scale bars, 100 μm. (B) Transwell assay (mean ± SD, n = 3). *P < 0.05. Scale bars, 50 μm. (C) The morphological changes of cells undergoing EMT. Scale bars, 50 μm. (D-E) Transwell assay (mean ± SD, n = 3). *P < 0.05. Scale bars, 50 μm. (F) The transfection efficacy detected by RT-qPCR and WB (mean ± SD, n = 3). *P < 0.05.

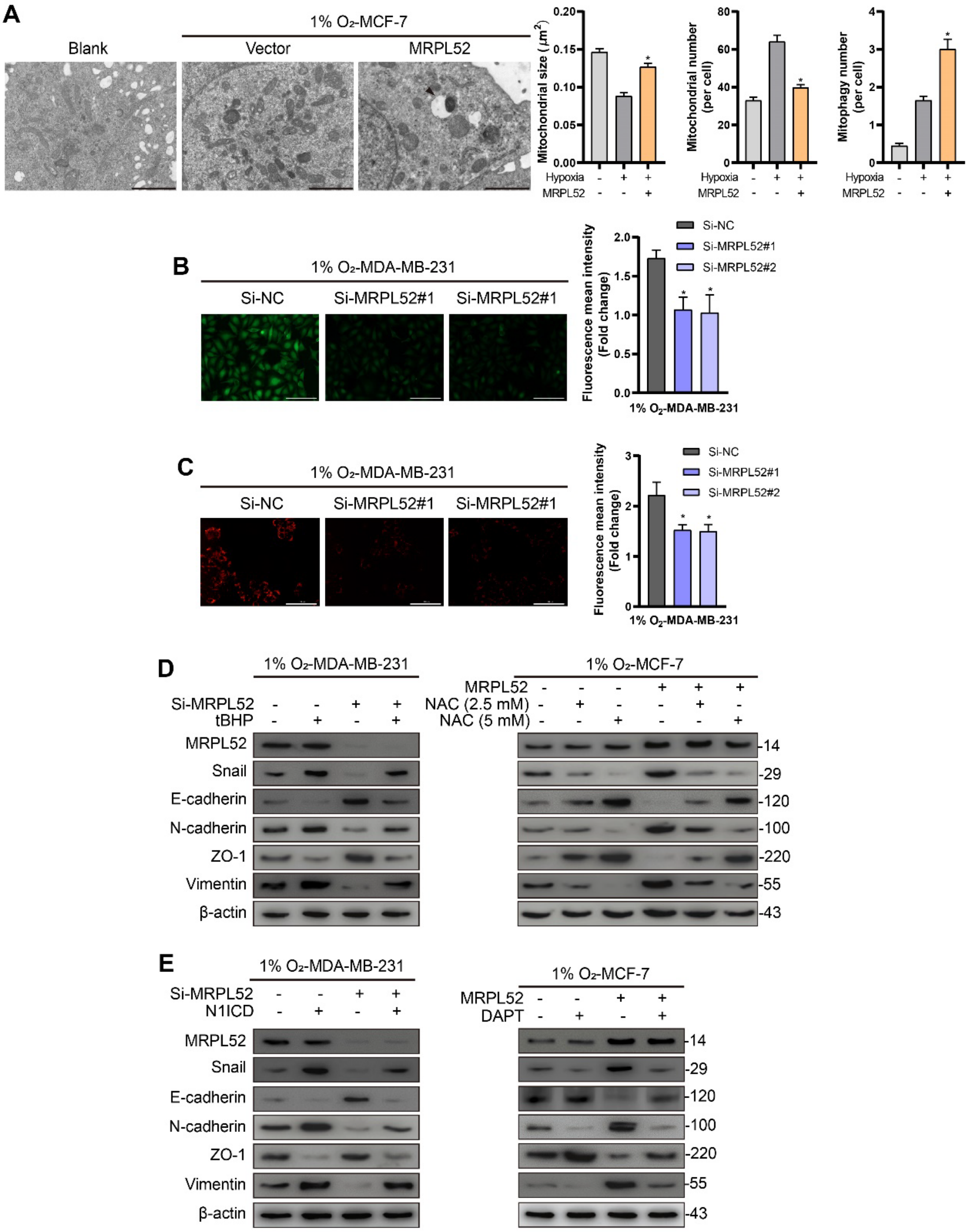

Figure S4 (A) Mitochondrial ultrastructure in cells under TEM. Black arrows point to mitophagy. Scale bars, 2 μm. (B) DCFH-DA and (C) MitoSOX fluorescence in cells measured by fluorescence microscopy. The fluorescence mean intensity of DCFH-DA and MitoSOX represent cytosolic ROS and mitochondrial ROS, respectively (mean ± SD, n = 3). *P < 0.05. Scale bars, 100 μm. (D-E) Cells were preincubated with tBHP (200 μM) for 4 h; or with NAC (2.5 or 5 mM) for 1 h. 10 μM DAPT was added into culture medium of cell for 24 h to inhibit Notch signaling. WB for detecting the expression levels of MRPL52, Snail, E-cadherin, N-cadherin, ZO-1 and Vimentin in MDA-MB-231 and MCF-7 cells exposed to 1% O_2 with treatments as indicated.

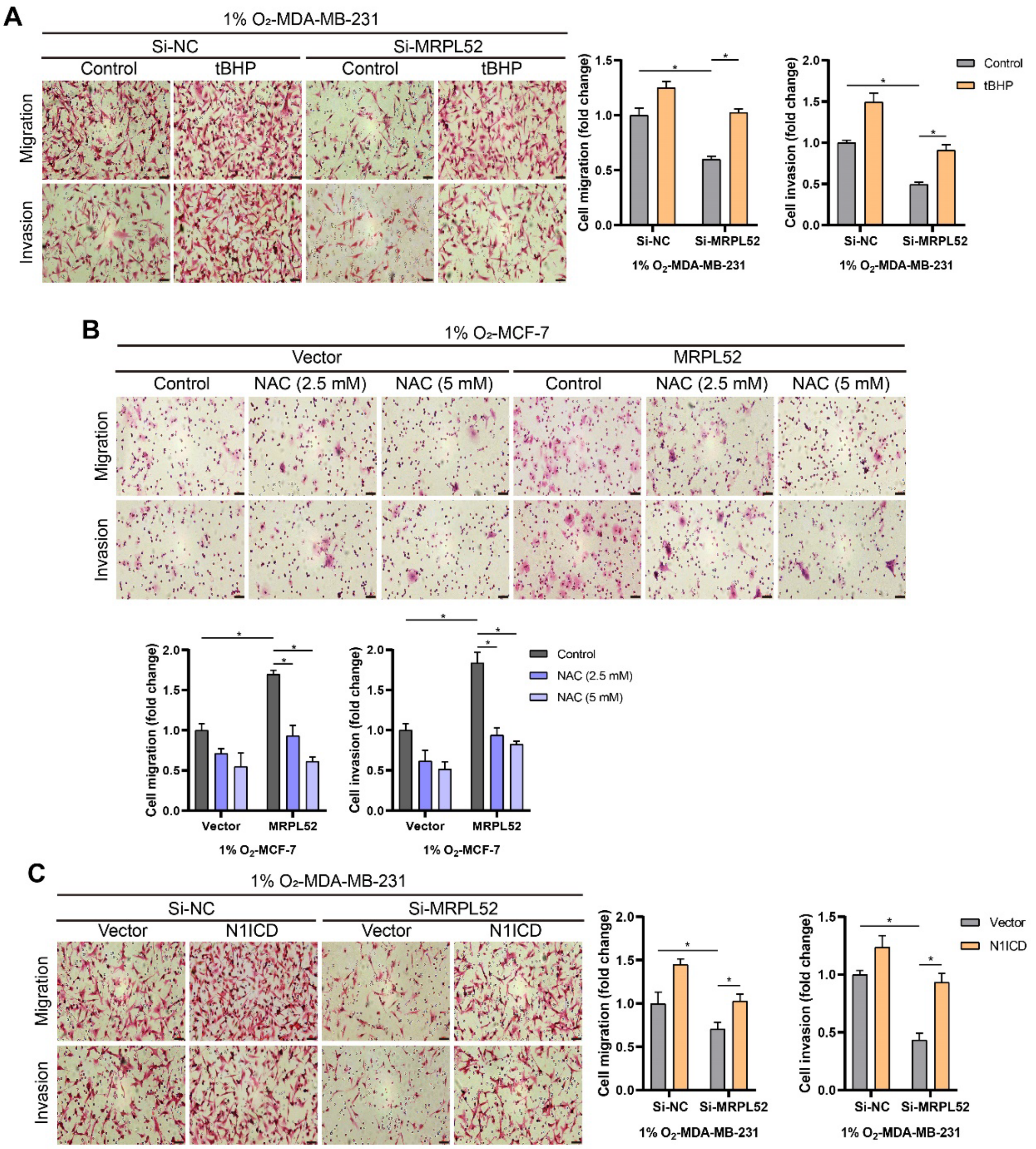

Figure S5 (A-C) Transwell assay (mean ± SD, n = 3). *P < 0.05. Scale bars, 50 μm.

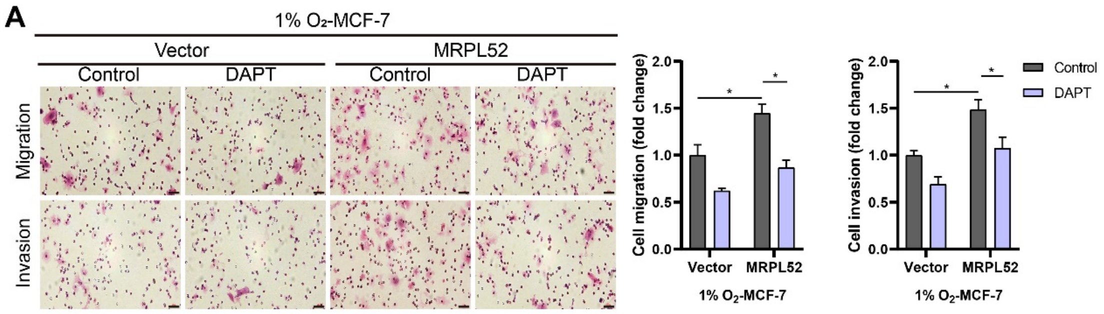

Figure S6 (A) Transwell assay (mean ± SD, n = 3). *P < 0.05. Scale bars, 50 μm.
